# Interactive music composition driven by feature evolution

**DOI:** 10.1186/s40064-016-2398-8

**Published:** 2016-06-22

**Authors:** Maximos A. Kaliakatsos-Papakostas, Andreas Floros, Michael N. Vrahatis

**Affiliations:** Department of Music, Aristotle University of Thessaloniki, 57001 Thessaloníki, Greece; Department of Audiovisual Arts, Ionian University, 49100 Corfu, Greece; Department of Mathematics, University of Patras, 26110 Patras, Greece

**Keywords:** Evolutionary music composition, Interactive music composition, Feature-based music composition, Particle swarm optimisation, Genetic algorithms

## Abstract

Evolutionary music composition is a prominent technique for automatic music generation. The immense adaptation potential of evolutionary algorithms has allowed the realisation of systems that automatically produce music through feature and interactive-based composition approaches. Feature-based composition employs qualitatively descriptive music features as fitness landmarks. Interactive composition systems on the other hand, derive fitness directly from human ratings and/or selection. The paper at hand introduces a methodological framework that combines the merits of both evolutionary composition methodologies. To this end, a system is presented that is organised in two levels: the higher level of interaction and the lower level of composition. The higher level incorporates the particle swarm optimisation algorithm, along with a proposed variant and evolves musical features according to user ratings. The lower level realizes feature-based music composition with a genetic algorithm, according to the top level features. The aim of this work is not to validate the efficiency of the currently utilised setup in each level, but to examine the convergence behaviour of such a two-level technique in an objective manner. Therefore, an additional novelty in this work concerns the utilisation of artificial raters that guide the system through the space of musical features, allowing the exploration of its convergence characteristics: does the system converge to optimal melodies, is this convergence fast enough for potential human listeners and is the trajectory to convergence “interesting’ and “creative” enough? The experimental results reveal that the proposed methodological framework represents a fruitful and robust, novel approach to interactive music composition.

## Background

Music is an expression of human creativity with widely-explored structural characteristics that are associated with the concept of “music aesthetics”, which describes the effect of music to humans. Music aesthetics are subjective, since each individual listener is differently affected by music. Recent advances in artificial intelligence and evolutionary computation have allowed the creation of computational algorithms that exhibit the spontaneity of human creativity, combined with the rule-based expression of musical form and structure. These algorithms pertain to the scientific topic named as “computational creativity”, which has been increasingly expanding the last years.^1^ Several evolutionary methodologies have been created that compose music according to stylistic constraints (Manaris et al. 2007), demarcated by evolutionary fitness norms in the form of aesthetically meaningful music features. These composition techniques will be hereby referred to as “*feature-based*”, since the fitness of their incorporated evolutionary algorithms depends on a set of targeted music feature values.

Concerning the subjectivity in the automatically composed music, the utilisation of “*interactive*” evolutionary algorithms appears advisable, since the fitness evaluation is performed by the human user in the form of melody rating and/or melody selection. Consequently, the evolution of compositions in this case aims towards generating music that is more pleasant to the listener. However, interactive evolutionary systems suffer from a fundamental drawback which counteracts the potential of evolutionary computation. This drawback is the *user fatigue* (Takagi 2001), which concerns the inability of human users to undergo vast amounts of rating (applying numeric values) or selection (selection/rejection of good/bad products) simulations. Therefore, the population sizes and the generation numbers are drastically deteriorated, neutralising the immense evolutionary dynamics.

The paper at hand introduces the combination of *interactive* and *feature-based* music composition in terms of an interactive system that exploits the merits of both compositional methodologies. The system is implemented in two levels: (a) the higher level, which incorporates the *particle swarm optimisation* (PSO) algorithm and a proposed variant, that evolves music features according to the feedback received by the user, thus realising interaction and (b) the lower level, where an evolutionary music composition scheme based on genetic algorithms (GA) composes music according to the features provided by the higher level. Under this implementation scheme, a population of features derived by the higher level is transformed into a population of melodies in the lower level and the ratings provided by the user to the composed melodies serve as fitness values of the features that these melodies represent.

The assessed measurements of the proposed system’s performance indicate a fast convergence to the user’s *subjectively optimal* melodies. However, the notion of *subjective optimality* is treated with *scepticism* in this paper: we do not necessarily argue that the currently examined system is indeed able to produce music that will please any user. The argument made in this paper is that the proposed methodology works as good as accurately

1. the utilised (higher level) features describe musical value and
2. the utilised (lower level) evolutionary composition system composes music according to these features.

Thereby, we do not necessarily claim—or, in any way, we do not examine—the efficiency of musical features per se in capturing subjective preference of human users. This study focusses on the following question: “given a set of efficient features that describe music quality, can a system be produced that uses these features for generating good quality music?” Therefore, the nature of the experimental processes that are meaningful in the context of this work is objective. A subjective assessment would not provide something fruitful, since the question does not concern how efficient the currently utilised features are, but how efficiently any given feature space is traversed by the proposed two-level evolutionary scheme.

In a future work, thorough subjective experimental evaluation of the proposed methodology will allow to address questions related to how different groups of human users reflect on such systems. For instance, would musicians consider using such a system for enhancing their creativity by manipulating the rating process so that unforeseen musical results emerge? Would non-musicians conceive this system as a tool for expressing their creativity by enabling them compose music according to ratings? In order to obtain meaningful answers to such human-oriented questions, extensive experimental analysis is required that places the focus on aspects of human perception rather than the issue of compositional convergence addressed in this paper.

In order to provide objectively admissible results we introduce a novel assessment methodology, which employs artificial, non-human raters. Compared to human listeners that may not be sure about which melody they considered pleasant at any given moment, an artificial rater may be constantly targeted to a specific melody, by providing higher ratings to melodies that assimilates it. Through this procedure, not only the “convergence” capabilities of the system are evaluated, but also the potential variability of the melodies throughout iterations is estimated. Furthermore, the weaknesses of the system are exposed by measuring its efficiency according to each musical feature separately, allowing for assumptions about potential future improvements.

The rest of the paper is organised as follows. “Literature overview and motivation” section provides a literature review over the descriptive quality of musical features and feature-based and interactive/evolutionary composition methodologies. A detailed description of the methodological context that this paper presents is provided in “Methods” section. The experimental methodology and the obtained results are presented in “Experimental methodology, results and discussion” section, where the concept of automatic raters is also introduced. Through the automatic raters, an objective assessment of the system’s convergence capabilities is attempted, along with the melodic diversity estimation that the system is able to produce. Furthermore, this section provides some insights about the weaknesses of the proposed system, leading to assumptions about its potential improvement. Finally, the paper concludes in “Conclusions” section.

## Literature overview and motivation

This work is motivated by the progress made in the hitherto separate fields of feature and interactive-based music composition. Evolutionary algorithms have enabled both researchers and artists to compose music with specific, target characteristics expressed as sets of “musical features” that are able to describe music qualitatively. The potential of these features towards identifying musical characteristics has been demonstrated over the last decade by feature-based pattern recognition approaches that have been successfully employed to identify several musical attributes, like the composer (Purwins et al. 2004; Wolkowicz et al. 2008; Kaliakatsos-Papakostas et al. 2010, 2011) or the musical style and genre (Kranenburg and Backer 2004; Mckay and Fujinaga 2004; Hillewaere et al. 2009a, b; Herremans et al. 2015). Furthermore, the incorporation of features that focus on mathematical measures of complexity has allowed the aesthetic characterisation of music, producing models that simulate how humans perceive music (Shmulevich et al. 2001; Madsen and Widmer 2007), leading also to models of subjective preference (Manaris et al. 2002, 2005; Machado et al. 2003; Hughes and Manaris 2012). The information capacity of these features has allowed the development of evolutionary systems that automatically compose music in a “supervised” manner. Such systems employ a evolutionary schemes, with fitness criteria defined as fixed target values among the aforementioned features and compose music with certain stylistic or aesthetic content, under the “supervision” of these features. Examples of evolutionary techniques with fitness based on complexity-based music features, can be found in Manaris et al. (2007), Alfonseca et al. (2007), Manaris et al. (2011), while systems that utilize musical-oriented target features were developed in Papadopoulos and Wiggins (1998), Biles (2002), Özcan and Ercal (2008), Matic (2010), Donnelly and Sheppard (2011), Herremans et al. (2015), Hofmann (2015).

Alternative evolutionary “supervised” approaches have been proposed based on direct human guidance. In these cases, fitness evaluation on individual-melodies is accomplished by humans, who either assign the fitness values through a rating scheme (Unehara and Onisawa 2005; Fortier and Van Dyne 2011; MacCallum et al. 2012; Kaliakatsos-Papakostas et al. 2012d), or allow certain melodic individuals to reproduce through a selection–elimination scheme (Sánchez et al. 2007). These “interactive” composition methodologies have important assets and drawbacks in comparison to the feature-based ones. A major asset is that the evaluation of musical individuals is “guaranteed” to be aesthetically meaningful, since it is directly appraised by the aesthetic preferences of the human user. Contrarily, feature-based techniques rely on measurements over specific musical styles or genres, thus deteriorate the prospectives of radical novelty in compositions that emerged by human judgments. Specifically, feature-based systems may only compose music according to style or genre “templates”, regardless of the subjective musical directions that the user might have. The primary and decisive drawback of interactive systems however, is related to the potential of the evolutionary process per se: the users are not able to undergo vast amounts of hearing and rating (or selecting) sessions, since it takes a forbiddingly large amount of time to evaluate large populations of individual-melodies evolved throughout a large number of generations, leading to *user fatigue* that additionally increases the uncertainty in ratings or selection and consequently misleads the evolutionary orientation. Therefore, interactive methodologies can hardly exploit the full potential of the evolutionary processes, which mainly relies on the combination of diverse possibilities that are encompassed by large numbers of population members.

An evolutionary process assesses the fitness of individuals through their “phenotypical” appearance and improve the population characteristics through “genotypical” interventions. However, it is not guaranteed that small alterations in the genotype of an individual will lead to small alteration to the phenotype (fitness). Therefore, an evolutionary scheme that includes a great number of individuals that are evolved for a great number of generations, may potentially conclude to the fine-tuned genotypical combinations that produce the desired phenotypical appearance, through numerous trial-and-error simulations. This fact subtly introduces an additional drawback: a small alteration of a well-fit individual-melody, or a combination of two well-fit individuals, is not guaranteed to result in the production of well-fit individuals. Thereby, the user is expected to hear and rate purely-fit individuals even in a quite progressed stages of evolution, a fact that amplifies user fatigue and further deepens the unclarity in user ratings. The difference between genotypical and phenotypical expressions of sound/musical individuals has been examined in Kaliakatsos-Papakostas et al. (2012c). In contrast to the lack of correspondence between genotypical and phenotypical distance, neighbouring locations in the feature space describe musical excerpts that share “neighbouring” musical characteristics, a fact that is evident by the style, genre and composer classification accuracy reported by existing works mentioned in the first paragraph of this section.

The work at hand introduces a balanced fusion of the aesthetically meaningful interactive human rating and the exhaustive explorational capabilities of feature-based composition. The proposed system consists of two levels: the higher level that models the human preference, and the lower level, where music is composed according to the user’s preferences, as reflected by the features on the higher level. The higher level evolves music features and provides them to the lower level, which utilises evolutionary techniques to compose music according to these features. The human subject rates the melodies produced following his own aesthetic criteria. In contrast to the interactive methodologies already discussed, the listener actually rates the *features* that are responsible for the production of the respective melodies. The rating process thus allows the user to explore the *feature space* rather than “randomly” recombine, apply crossover and mutate musical genotypes. The term “randomly” above is used to express the fact that there is uncertainty in the effect that genotypical alterations have, since small changes on the genotypical level may result in vast changes on the phenotypical level.

The proposed approach incorporates the PSO algorithm on the higher level, whereas any heuristic alternative could have been used. The selection of PSO relies on two basic factors. Firstly, since the system is interactive, fast convergence is required, making PSO a prominent choice (Vesterstrom and Thomsen 2004). Secondly, the feature space incorporates neighbouring regions that encompass similar musical characteristics. Therefore, the circumscription of particle orbits *from* their current position *to* the best position is important, since the user expects to hear as less random melodic transitions between rating iterations as possible. The existence of the “cognitive best” and “social best” coefficients in the PSO algorithm ensures that the transitions between successive agent steps are locally coherent, leading also to more promising directions. These characteristics of PSO are presumed to improve the interactive experience of the user, partially alleviating the imposition of user fatigue.

Several aspects of the proposed methodology need to be investigated in order to evaluate the aforementioned assumptions. Firstly, an evaluation of its robustness has to be performed, by examining the ability to “converge” to pleasant features/melodies indicated by the user. Secondly, the characteristics of the composed music throughout the evolutionary steps have to be analysed, as expressed by their positions in the feature space. This analysis is important because of the interactive perspective of the system; it is important to assess the feedback that the system provides to the user by examining the diversity of the melodies presented after each rating. Finally, the cooperation between the higher and the lower level has to be examined in detail, in order to obtain insights about the strengths and weaknesses of the proposed cooperative architecture.

## Methods

**Fig. 1:**
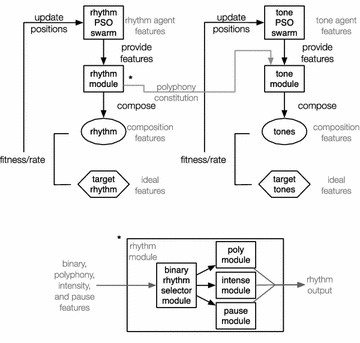
Block diagram of the proposed system

The developed system is designed to function according to the ratings provided by users—even though the experimental orientation does not include user-driven results. In a common scenarion, during evolution, each user listens to and subjectively rates four different evolving melodies in every rating round, based on two musical criteria: rhythm and tone. We have primarily investigated a single-valued rating scheme, i.e. rating how good is the melody, but it was rejected as it led to deadlock rating dilemmas: how should a listener rate a melody with, e.g. pleasant rhythm and unpleasant tone? On the other hand, “finer” rating subdivisions of multiple musical features (e.g. how good is chord-structure, rhythm syncopation, tonality balance) were found to be confusing for the mean user. Therefore, a rating scheme including two ratings for each melody, namely the “rhythm” and “tone”, was chosen. Figure 1 illustrates a block diagram of the proposed system. User rhythm and tone ratings are processed almost independently, except from some rhythmic constraints that are imposed on the tone generation process. The architecture of both parts is identical, with their higher levels incorporating PSO and the lower levels comprising a set of GA-based music generation modules. The higher level PSO employs agents that roam in a space of several music features, while the lower level GA schemes compose music by generating rhythmic and tonal sequences respectively, according to the feature combinations provided by each agent.

Two PSO swarms are used for describing rhythm and tonal features respectively, while each of these swarms comprises four agents. Each agent’s coordinates in turn, represent a set of rhythmic and tonal features respectively. Therefore, the location of an agent describes a unique feature combination that is rendered as specific rhythmic or tonal sequences respectively by the lower level GA modules. For example, the location of a rhythm agent signifies a certain rhythmic features combination, which is “delivered” to the underlying rhythm composition GA as guidelines to compose a rhythmic sequence. The same holds for the tone PSO agents and the underlying tone generation GA modules. In general, the position of a PSO agent constitutes the fitness criterion for the respective GA composition module, in a sense that the GA module aims at composing music (rhythmic or tonal sequences) with features that are as similar as possible to the ones dictated by the respective higher level PSO agent. Furthermore, each agent in the rhythm swarm is linked to an agent at the tone swarm, meaning that a melody is finally composed by combining the rhythmic and the tonal sequences of a certain pair of rhythm and tone agents. Besides, the tone GA composes a tonal sequence over a given rhythmic sequence, as described in “Evolutionary generation of melodies” section.

Each pair of rhythm and tone agents describes a melody. Thereby, the quadruples of rhythm and tone agent pairs are rendered as four melodies through the respective underlying GA modules. As mentioned previously, a listener subjectively rates these four melodies providing fitness evaluation to the respective rhythm and tone PSO agents. In the next rating round, the rhythm and tone agent pairs quadruples are evolved with a properly adjusted PSO agent location update rule presented in “PSO initialisation and evolution” section, moving to new positions that correspond to new music features (i.e. combinations of rhythmic and tonal features). In turn, the underlying GA modules compose music according to the updated agent locations/features, presenting four novel melodies to the user that constitute the melodic content of the new rating round. Through this iterative process, user’s ratings are expected to lead the PSO agent quadruples to feature regions that reflect pleasant music characteristics.

### Basic user-oriented assumptions

The aim of the proposed system is to facilitate user interaction. The following paragraphs focus on some basic assumptions about what a user would expect and tolerate from such a system. The experimental processes that are described later aim to monitor whether the system satisfies these assumptions. The basic assumptions presented in this section concern generally admissible facts about interactive music generation systems, while, as a novelty of this paper, the experimental results examine these assumptions without any subjective test: artificial raters are employed that simulate the behaviour of human users.

There are some aspects of interactive music evolution that impose convergence to “subjective optima” limitations. Due to the *user fatigue* that results from the constant human devotion to the task of listening and rating, the listener is expected to loose focus during the rating process, especially if she/he undergoes a large number of melodies in each rating round. This obviously increases the hazard of inconsistent or even contradicting ratings, misleading the system to non-optimal feature regions. Consequently, this would force the agents to roam the feature space without converging to certain melodic locations, creating an impression that the system does not provide any feedback to the user ratings, further amplifying the vicious circle of user fatigue. User fatigue does not solely depend on the number of melodies, but also on their duration. Since the melodies that the proposed system produces are about 15 s long (as described later in “Evolutionary generation of melodies” section), a collection of 4 melodies per rating round was considered as a satisfactory compromise between melodic diversity (considering also the initialisation procedure discussed in “PSO initialisation and evolution” section) and keeping the number of melodies as low as possible.

Independently of the number of melodies in each rating iteration, user fatigue is also expected to emerge at some point, since there is a generally admissible time limit that a human can listen to melodies carefully, no matter how interesting these melodies are. Moreover, since it is desirable to have gradual convergence to an optimal region in the feature space, all melodies that comprise consecutive quadruples are expected to become more similar as the iteration progresses. Hence, after a number of rating rounds, the user will be required to rate similar melodies, a fact that increases user fatigue and, consequently, inconsistent and/or contradicting ratings. The above mentioned two points highlight an additional constraint: *the system should be progressing towards better rated melodies swiftly, within a small number of rating rounds*. Similarly to the decision made about the number of melodies in each rating iteration, the estimation of a maximum number of rating rounds is also dependent on the expected duration of rating each quadruple. Since each melody is about 15 s long, each rating round (4 melodies) is expected to last about 1 minute, therefore allowing a rough estimation of 20 rating iterations (about 20 min) per user at maximum. Although the limit of evolutionary iterations is case-dependent, a human listener is anticipated to undergo a maximum limit of 10–20 iterations (Takagi 2001).

### PSO initialisation and evolution

The melodies in all rating rounds are four and, according to the aforementioned basic assumption, in parallel to their swift fitness convergence, they should encompass as diverse (and well fit) characteristics as possible, covering a range of features that is as wide as possible. Hence, the user should be offered a wide spectrum of pleasant potential melodic possibilities that would allow a more efficient and productive exploration of the overall music feature space. The required diversity of features is achieved through an initialisation scheme that guarantees that every pair of melodies has at least one pair of “sufficiently distant” features (rhythmic or tonal). The employed initialisation algorithm roughly loops through all the features, randomly divides the four agents in two pairs and for each pair it assigns two random feature values that are separated by a minimum preselected percentage of this feature’s range. For example, we may consider the polyphony mean feature, defined as the mean number of simultaneous notes per onset event. For the purposes of this work, this feature takes real values within [1, 5]. Therefore, if the least distance percentage is set to 80 %, then in one of the randomly selected pairs of agents, one agent would be assigned with a value $a = \text {random}(1,1+(1-0.8)(5-1))$, while the other with a value $b = \text {random}(a+0.8,5)$, where the function $y = \text {random}(x_1,x_2)$ returns a random real number in $[x_1,x_2]$. The same would be performed for the remaining two agents of the other pair. By employing a random agent pair selection, this initialisation scheme actually shuffles extreme music characteristics and randomly dispenses them among the four agents of the initial PSO populations. The limit used for the experimental results was 90 %, in order to achieve an extreme diversity among the four initial melodies.

The subsequent agents’ movement rules need also to be adapted in order to meet the constraints imposed by the interactive nature of the system. As discussed previously, the user anticipates that the system evolves melodies towards better ones swiftly and in accordance to the rates that she/he provides. Hence, the listener should feel that there is no lack of feedback from the system and that his rating is as meaningful as possible, in a sense that higher-rated melodies are less altered than lower-rated melodies. To this end, a novel variation of the PSO algorithm was formed, which encompasses information about the ratings (fitness) that a melody has been assigned, allowing the respective (rhythm or tone) agent to move faster or slower in correspondence to its rating.

The first formalisations of PSO were provided in Kennedy and Eberhart (1995), where the position of each agent was updated for each dimension based on the position of the agent’s former optimiser value, as well as on the position of the global swarm optimiser value. The new position of each agent is computed as the sum of its previous position with a quantity that is estimated according to several factors, depending on the PSO variant, which incorporate the agent’s and the swarm’s best positions. Specifically, the *i*-th agent is initialised in a position $\vec {x_i}(0)=(x_{i1}(0),x_{i2}(0),\dots , x_{iD}(0))^{\top }$ and utilising the inertia weight (Shi and Eberhart 1998a, b) PSO variant, the position of the agent in every time step is updated by

1 $$v_{ij}(t+1)= w\ v_{ij}(t) + c_p R_p (p_{ij}(t)-x_{ij}(t)) + c_g R_g (p_{gj}(t)-x_{ij}(t))$$

2 $$x_{ij}(t+1)= x_{ij}(t) + v_{ij}(t) ,$$

where $i = 1,2,3,4$, $j = 1,2,\dots , D$, *w* is the inertia factor, $c_p$ and $c_g$ are the biases towards the personal best position of the agent and the global best of the swarm and $R_p$ and $R_g$ are random numbers chosen uniformly in [0, 1]. The quantity in Eq. 1 is called the “velocity” of the agent and the location update in each dimension is performed through adding the respective velocity coefficient to the agent’s current location, as demonstrated in Eq. 2. For a thorough review of PSO algorithms, the interested reader is referred to Parsopoulos and Vrahatis (2010). It is also parenthetically mentioned that several works have utilised swarm intelligence [inspired by the “*boids*” algorithm (Reynolds 1987, 1988)] for music composition (Blackwell and Bentley 2002; Blackwell 2003, 2007; Jones 2008) and sound synthesis (Blackwell and Young 2004; Blackwell 2008; Wilson 2008).

For the presented system, a modified formula for the computation of velocity has also been tested, which encompasses some characteristics that improve the overall interactive experience. In particular, a coefficient is added which introduces de facto noise to the computation of velocity. In Eq. 1, after the first rating round, the agent that carries the best rating remains unaltered, since all the products on the right side of the equation have at least one zero-valued term. Specifically, $v_{bj}(0)=0$, $p_{bj}(0)=x_{bj}(0)$ and $p_{gj}(0)=p_{bj}(0)=x_{bj}(0)$, where $b\in \{ 1,2,3,4 \}$ is the index of the best rated agent in the first rating round and $j = 1,2,\dots , D$. Therefore, as long as the initially higher rated agent remains the higher rated in subsequent rating rounds, the characteristics of the best melody will remain unaltered, providing the user with an essence that the system does not evolve according to her/his ratings. For instance, if a user provides a maximum rating of 3 out of 10, then the characteristics of the melody that has been rated with 3 will remain unaltered, a fact that is not justified by its overall low rate.

On the other hand, since the number of iterations is expected to be small (earlier roughly computed around 20) and the number of agents is also restrictive (four melodies per rating round) the movement of each melody throughout the iteration rounds has to be as deliberately calculated as possible. Thus, the melodies with higher ratings for rhythm or tone should be wandering into the respective space with more “self-confidence” than the ones that obtain lower ratings. The term “self-confidence” expresses the notion of increased bias towards the personal best. Contrarily, a lower rated agent should steer more decisively towards the “safer” global best. To this end, the novel velocity update formula does not incorporate the constant $c_p$ and $c_g$ values; instead, these values are adjusted according to the current rating of a particular agent. For facilitating the reference to this modified PSO, the term *rating-based* PSO (*r*-PSO) is employed hereby. Similar modifications, which utilize the fitness of an agent to determine its location update potential have recently been proposed (Yang et al. 2007; Akbari and Ziarati 2011).

The velocity update formula for the *r*-PSO becomes

3 $$\begin{aligned} v_{ij}(t+1)&= w\ v_{ij}(t) + f_p(\beta _{c}) c_p R_p (p_{ij}(t)-x_{ij}(t)) \nonumber \\&\quad + f_p(\beta _{c}) c_g R_g (p_{gj}(t)-x_{ij}(t)) + f_r(\beta _{c}) \vec {R}_{j} , \end{aligned}$$

where $\vec {R}$ is a vector of uniformly selected random numbers within a proportion of the search space, $f_p(x)$, $f_g(x)$ and $f_r(x)$ are functions that receive the current rate of the agent ($\beta _{c}$) and are expressed by

4 $$f_p(\beta _{c})= \frac{\beta _{c}-\beta _{w}}{\beta _{b}-\beta _{w}+1} ,$$

5 $$f_g(\beta _{c})= \frac{\beta _{b}+1-\beta _{w}-(\beta _{c}-\beta _{w})}{\beta _{b}-\beta _{w}+1} ,$$

6 $$f_r(\beta _{c})= \frac{\beta _{b}+1-\beta _{w}-(\beta _{c}-\beta _{w})}{\beta _{b}-\beta _{w}+1} ,$$

where $\beta _{w}$ is the worst rating in the current rating round and $\beta _{b}$ is the global best rate in all previous rating rounds in the simulation. Through Eq. 4, the magnitude of the “cognitive” product ($c_p R_p (p_{ij}(t)-x_{ij}(t))$) increases as the rate of the agent ($\beta _{c}$) increases, with a minimum “cognitive confidence” of zero being achieved for the lowest rated agent ($\beta _{c}=\beta {w}$). Contrarily, the coefficients of the “social” product ($c_g R_g (p_{gj}(t)-x_{ij}(t))$) decreases as the rate of the agent increases. The modification in the velocity is not necessarily targeted towards improving the effectiveness of the PSO swarms in terms of fitness, but mainly towards engaging the user with diverse musical material that is evolving rationally according to her/his rating, throughout the rating iterations. Nonetheless, as the results in “Experimental methodology, results and discussion” section indicate, not only a diversity in the melodies throughout and within each rating round is achieved, but also the overall optimisation effectiveness of the system is slightly improved.

### Evolutionary generation of melodies

Under the proposed approach, automatic music composition algorithms generate melodies that comply with the constraints reflected by the features carried by each agent. Both rhythm and tone generation rely on GAs, through which binary or integer sequences are evolved. The evolution of these sequences is based on their interpretations to melodies and their consequent fitness evaluation according to a set of 39 music features (22 rhythmic and 17 tonal). In the context of this work, modified version of feature-based evolutionary algorithms presented in the literature for music composition have been developed. However, since the scope of the paper concerns the study of the convergence behaviour, a detailed description of the lower level composition algorithms is omitted.

The utilisation of evolutionary algorithms for the generation of rhythmic sequences has been previously explored for both percussive (Ariza 2002; Eigenfeldt 2008; Sioros and Guedes 2011; Yamamoto et al. 2012; Kaliakatsos-Papakostas et al. 2013) and pitched (Horowitz 1994; Kaliakatsos-Papakostas et al. 2012b) instruments. In the majority of these cases rhythms are derived that encompass certain characteristics, by fostering promising generations of rhythmic sequences through evolutionary processes. The key notion to the evolution of rhythms is the definition of proper fitness criteria that accurately describe the quantitative characteristics of the desired target rhythm. Therefore, the evolutionary approach to automatic rhythm composition incorporates a functional modeling of rhythmic sequences in the form of evolvable entities, in combination with a set of rhythmic features that operate as fitness criteria, driving the evolution to rhythms that adhere to certain qualitative characteristics.

The generation of rhythms in the context of the proposed system incorporates not only information about the time that a note event will happen, but also the polyphony and the intensity of this event, as well as information about pauses. The polyphony of an event indicates the number of notes that are simultaneously played. The intensity describes the loudness at which the notes during an onset event are heard. The pauses denote silence events, where all remaining notes are interrupted until a new note onset event occurs. The rhythm module employed here incorporates four submodules, which manage all the aforementioned rhythmic properties, namely the *binary*, the *polyphony*, the *intensity* and the *pause* submodules. These submodules produce rhythms according to the 22 rhythmic features provided by the respective agents in the rhythm swarm. From these 22 features, 5 are addressed to the binary, 6 to the polyphony, 6 to the intensity and 5 to the pause submodules.

**Fig. 2:**
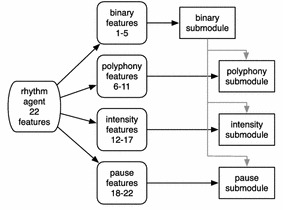
Block diagram of the rhythm module

The block diagram of the rhythm module is shown in Fig. 2. All melodies considered here are 4 measures with 4/4 time signature and an analysis of 16-ths, which are composed separately and then merged. The *binary* submodule produces binary sequences, with the digit 1 denoting an onset and 0 denoting a “no action” rhythm event. Since each measure is composed of 16 digits, the number of different binary rhythms is $2^{16}$. Therefore, when an agent requests a rhythm with certain binary characteristics (through the 5 features that describe it) the binary submodule searches throughout all $2^{16}$ binary rhythms and returns the ones that are more suitable. The binary submodule does not incorporate any evolutionary architecture, since the binary rhythm search space is small.

The *polyphony* and *intensity* submodules on the other hand, require the utilisation of evolutionary algorithms since the search space is overwhelmingly large. These submodules are dependent on the output of the binary submodule. Specifically, they isolate the onset events described in the binary submodule (occurrences of digit 1) and assign to them an integer within [1, 5] for the polyphony and [50, 120] for the integer submodules. The values [1, 5] denote the existence of 1–5 simultaneous notes, while the [50, 120] values denote MIDI velocities (intensities) ranging from 50 to 120. Proper integer combinations are examined using GA. These submodules produce suitable integer sequences that satisfy the demands of a rhythm agent, reflected in the features it carries (specifically, from feature 6–11 for polyphony and 12–17 for intensity submodules). Finally, the *pause* submodule locates positions of possible pauses, which are the no-onset (mapped to the 0 digit) of the binary submodule. By utilizing GA, the pause module examines the suitability of different pause scenarios by comparing the pause features they produce with the ones carried by the guiding rhythm agent (features 18–22).

**Fig. 3:**
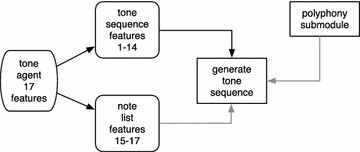
Block diagram of the tone module

The block diagram of the tone module is depicted in Fig. 3. This module utilizes GA to construct integer sequences in [36, 120], which are subsequently mapped to MIDI notes. The length of the integer sequences depends on the number of notes that the melody’s measure includes, which is provided by the polyphony rhythm vector. The notes for the tone sequences generated by the GA are selected from a note list formed using 3 criteria: music scale, lowest note and octave range. These quantities are provided to the tone module as numeric values by the tone agent’s coordinates indexed from 15 to 17 (from the 17 total features that the tone agent provides). The agent’s coordinates from 1 to 14 are the agent’s compositional guidelines, which are provided to the tone module as tonal features, on which the fitness evaluation is based. These features incorporate information that describe the complexity of the pitch class profile distribution through Shannon Information Entropy (Shannon 2001), descriptive statistics of the note and pitch class transitions’ distributions, the percentages of ascending, descending and constant transitions (Coca et al. 2010) and the chord potentiality of note clusters. A similar approach for automatic generation of tones has been followed in Kaliakatsos-Papakostas et al. (2012a).

## Experimental methodology, results and discussion

The lower level rhythm and tone generation modules may incorporate errors towards the production of rhythmic and tonal sequences, since it is not ensured that they will be capable to absolutely comply with the guidelines provided by the respective PSO agents (achieve perfect fitness). Thereby, a melody that is composed under a PSO agent features’ guidelines may not necessarily reflect these features exactly. Additionally, the position of an agent in the feature space may provide the underlying submodules with contradicting features, which may not be satisfied by definition, e.g. an agent may require 60 % ascending note intervals and at the same time 70 % descending note intervals, resulting to an unachievable sum of 130 %. Moreover, the ratings provided by a human listener may incorporate uncertainty and noise, i.e. the user may not feel absolutely confident about the aesthetic quality of a melody.

The presented combined bilevel evolutionary scheme models the above errors (or noise) in both the lower and higher level music generation modules, taking into account a) the potential incapability of the composition level to absolutely comply with the feature level agents’ guidelines and b) the instability of human ratings. Therefore, the system’s evaluation focuses on the convergence behaviour under the circumstances that incorporate the aforementioned efficiency impediments. The former impediment is endogenous to the system. The latter one depends on subjective factors and may not be directly quantified; hence they should be modeled by considering very general admissions, as discussed in “Assessment of performance through automatic raters” section.

By considering the potential composition inefficiency of the system, several questions may raise: does the system converge to optimal melodies? If the system does converge, how may the convergence characteristics be quantified? If there are impediments in convergence, which are the causes? The experimental results reported in this section provide answers to these questions by employing four PSO parameter *setups*, two of which pertain in the standard PSO, while the remaining two follow the *r*-PSO velocity update scheme. Although several values of *w*, $c_p$ and $c_g$ have been examined for both PSO and *r*-PSO cases, results are reported for the following representative PSO parameter setups:

1. setup-1 ($\hbox {S}_1$): PSO velocity update, $w=0.3$, $c_p=1$ and $c_g=1$,
2. setup-2 ($\hbox {S}_2$): PSO velocity update, $w=0.3$, $c_p=0.2$ and $c_g=1$,
3. setup-3 ($\hbox {S}_3$): *r*-PSO velocity update, $w=0.3$, $c_p=1$ and $c_g=1$ and
4. setup-4 ($\hbox {S}_4$): *r*-PSO velocity update, $w=0.3$, $c_p=0.2$ and $c_g=1$.

For both *r*-PSO setups, the random perturbation vector, $\vec {R}$, takes values within a 10 % margin of the respective dimension’s magnitude.

### Assessment of performance through automatic raters

Answering questions that incorporate convergence by subjective human ratings tests is challenging. Testing whether the system “converges” incorporates the exact allocation of a set of “*ideal features*” that the system will pursue to capture. The human users may not be certain about the ideal features that they require from a music piece. This fact does not only rely on the subjectivity of each listener to music pleasantness, but also to the conditionality that this pleasantness is actually expressed. For example, the shadowgraph of some potential “ideal feature” that a human rater may have in mind at some point during the simulation, may be influenced by a melody that she/he hears during a rating round. Thereby, these “ideal features” are expected to steer towards different musical directions throughout the rating simulation. This steering in the preferred features that a human rater may experience is a desirable effect of human cognition and creativity; however the aim of this research is to quantify the extent at which the system is able to *follow* the directions provided by a user, even if these directions change within simulations.

To this end, a “test-tube” experimental methodology is formulated, where the rates are provided by *automatic raters*. These raters simulate some basic rating characteristics of human raters, but provide ratings in accordance to the fixed set of features, called the “ideal features”. In contrast to a human user, the ideal features of the automatic raters remain fixed throughout every rating simulation. The ability of the system to move towards these features is scrutinised by employing several rating simulations with several “almost” random ideal features, carried out by automatic raters with different rating characteristics. The term “almost” is utilised in a sense that these ideal features should describe a music piece that is potentially realizable, therefore an absolutely random procedure would produce controversial and mutually-rejecting features (like the aforementioned example of the unrealisable 130 % percentage of ascending and descending intervals). Therefore, the ideal features that each automatic rater encompasses are the ones of a piece composed by a random selection of features. The ideal features could also be extracted from well-known music compositions; however, this idea was rejected in order to avoid restricting the considered compositional scope.

In order to construct an ideal features’ set for a rating simulation a random point in the feature space is selected which functions as the compositional guidelines to the music composition modules. The feature combination represented by this point in the feature space could incorporate controversial features, as described earlier. Nevertheless, the melody that is finally composed by the music composition modules incorporate features that belong to a melody by definition. Thus, this process ensures that the ideal features of the automatic raters in each rating simulation are potentially realisable. The automatic raters are guaranteed to have a fixed set of target ideal features according to which they provide their ratings. It is assumed that a melody will be rated with a higher value, if its features are closer to the ideal features of the rater. By denoting the ideal features of a rater in a simulation as $\vec {f_{*}^{r}}$ for the rhythm features and $\vec {f_{*}^{t}}$ for the tone features, the rate that the automatic rater will assign to a melody with features $\vec {f_{c}^{r}}$ and $\vec {f_{c}^{t}}$ is inversely related to the respective distances:

7 $$q_{r}= \left\| \vec {f_{*}^{r}} - \vec {f_{c}^{r}} \right\| _2 ,$$

8 $$q_{t}= \left\| \vec {f_{*}^{t}} - \vec {f_{c}^{t}} \right\| _2 ,$$

while their connection to the final rating is described later, by Eq. 9. Additionally, to simulate the introduction of human ratings uncertainty, a random value is also added, which is potentially allowed to violate the better fitness–better rate principle.

**Fig. 4:**
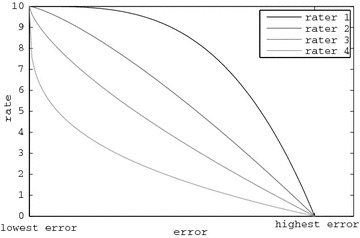
Illustration of the assigned automatic raters’ rating profiles

For the artificial raters several “rating profiles” were modeled, employing different criteria towards how high a rating describes a good melody. For instance, a strict rater may provide a higher rate of 5 to the melodies he finds most appealing. Contrarily, a less strict rater may rate with 9 some appealing melodies. Since the PSO and melodies evolution relies on ratings, the convergence of the system is examined in accordance to four different rating profiles, which are calibrated to model a variety of potential users from non-strict to strict. The fitness-to-rating correspondence of the four rating profiles that are utilised for the experimental results is depicted in Fig. 4. These rating curves assign a rate ($\beta _{x}$) to a fitness value ($q_{x}$) for a rhythm ($x=r$) or tone ($x=t$) agent using the equation:

9 $$\beta _{x} = 10-10 \left( \frac{q_{x}-l_{x}}{u_{x}-l_{x}} \right) ^\alpha + \xi _{\beta } ,$$

where $l_{x}$ and $u_{x}$ are the lower and upper bounds of fitness for rhythm ($x=r$) and tone ($x=t$) and $\xi _{\beta }$ is a random number in $[-0.5,0.5]$ that simulates human rating uncertainty. The parameter $\alpha$ defines the “strictness” of the artificial rater, with higher values denoting a less strict rater. The values of $\alpha$ depicted in Fig. 4 are 3.3, 1.3, 0.7 and 0.3, which are also the values of the four automatic raters employed in this work.

The underlying music composition modules that have been formulated for the bottom level of the proposed system are hardly capable to compose melodies that perfectly match the features requested by the agents. Therefore, the $l_{x}$ values are set to a near-minimum error quantity below which the rhythm and tone modules can hardly reach. Accordingly, the value of $u_{x}$ is set to a near-maximum error quantity of the rhythm and tone modules. After thorough experimentation, and by considering a rating scale in [0, 10], the values that have been selected for the respective modules are the following: $l_{r}=2$, $l_{t}=1$, $u_{r}=13$ and $u_{t}=8$. It should be noted that these values are system-dependent and consequently, apply to the music composition modules of the presented system. For the experiments that follow, the four rater profiles considered are the following:

1. rater-1 ($$\hbox {R}_1$$): $$\alpha = 3.3$$,
2. rater-2 ($$\hbox {R}_2$$): $$\alpha = 1.3$$,
3. rater-3 ($$\hbox {R}_3$$): $$\alpha = 0.7$$ and
4. rater-4 ($$\hbox {R}_4$$): $$\alpha = 0.3$$.

For each rater and setup combinations the results of 50 rating simulations were examined and are presented next.

### Fitness convergence analysis

The primary concern is to examine whether the system presents overall converging behaviour or not. System convergence can be expressed as the reduction of distances between the rhythm and tone features of the composed music ($\vec {f_{c}^{r}}$ and $\vec {f_{c}^{t}}$ respectively) and the respective ideal features ($\vec {f_{*}^{r}}$ and $\vec {f_{*}^{t}}$) that the automatic rater demonstrates throughout the rating rounds. The features of the composed music ($\vec {f_{c}^{r}}$ and $\vec {f_{c}^{t}}$) depend on the features provided by the respective rhythm and tone PSO agents ($\vec {f_{a}^{r}}$ and $\vec {f_{a}^{t}}$); specifically $\vec {f_{a}^{r}}$ and $\vec {f_{a}^{t}}$ are provided as the target features for the underlying music composition modules. As previously mentioned, these modules would function “perfectly” if they were able to compose music that adheres exactly to $\vec {f_{a}^{r}}$ and $\vec {f_{a}^{t}}$, thus it would hold that $\vec {f_{c}^{r}} = \vec {f_{a}^{r}}$ and $\vec {f_{c}^{r}} = \vec {f_{a}^{t}}$. In this ideal scenario the overall system’s convergence would be trivial: the system would convergence if the higher level PSO algorithm converged. In this case, the overall converging behaviour of the system would absolutely depend on the parameter setup of the PSO modules.

Nevertheless, the underlying composition modules can hardly compose music precisely according to the music requested by the respective PSO agent’s features. It is thus expected that the features of the composed music, $\vec {f_{c}^{r}}$ and $\vec {f_{c}^{t}}$, will be similar but not identical to the requested features, $\vec {f_{a}^{r}}$ and $\vec {f_{a}^{t}}$. Therefore, the overall system’s convergence does not only depend on the top-level PSO convergence, but it is also affected by the effectiveness of the underlying music composition modules, which is analysed in “Adaptation of specific music features” section in detail. Additionally, even if the system converges, there is a crucial matter of how fast this convergence occurs, since the convergence rate is crucial for the effectiveness of the system’s *interactivity*. For example, slow convergence could distract the user’s attention, providing him with an essence that the produced output does not change according to hers/his directions. It would be thus substantial to examine the system’s convergence within the time span that a user would undergo without the imposition of fatigue. Therefore, as discussed in “Methods” section, a maximum number of 20 rating iterations was considered.

**Fig. 5:**
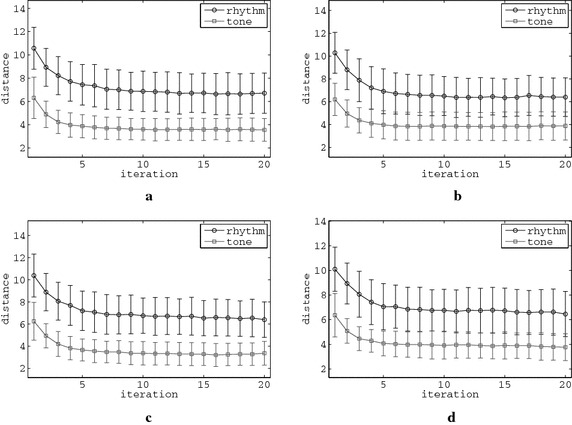
*Error bars* of total fitness for all 50 simulations of some representative PSO setups of rhythm and tone generating modules, for all ARs. **a** $\hbox {R}_2$-$\hbox {S}_1$. **b** $\hbox {R}_2$–$\hbox {S}_2$. **c** $\hbox {R}_2$-$\hbox {S}_3$. **d** $\hbox {R}_2$-$\hbox {S}_4$

Figure 5 illustrates the basic statistical convergence behaviour of the system, based on the mean value of errors of all melodies in every rating round, i.e. the distances between the music composed by the guidance of all four PSO agents and the ideal features calculated as:

10 $$\mu = \frac{1}{4}\sum _{i=1}^{4} \left\| \vec {f_{c,i}^{x}}-\vec {f_{*}^{x}}\right\| _2 ,$$

where *i* is the PSO agent’s index and $x \in \{ r,t \}$. The error bars appeared in the above figure indicate the mean values and the standard deviations of the aforementioned mean distances in each iteration for all 50 simulations. These illustrations concern some representative artificial raters and setups, while the respective illustrations for the remaining raters and setups exhibit similar characteristics, i.e. all errors reduce to a minimum value, as also discussed later in Table 1. Thereby, the mean error for all agents in both the rhythm and the tone PSO swarms is gradually reduced, indicating an asymptotical convergence of all four PSO agents for both rhythm and tone swarms to a minimum value. Some aspects of individual agent convergence behaviour are further addressed in “Music features convergence analysis” section.

The statistical improvement in the mean fitness of all four composed melodies in a rating round is also demonstrated in Table 1, where several statistical values signify that the convergence to the “ideal features” is within the assumed limit of 20 rating rounds. The first and second columns of this table demonstrate the mean and standard deviation values (in parentheses) of the mean error described by Eq. 10 among all simulations during the first and last PSO iteration respectively for all the artificial raters and PSO setups. The relative improvement of this error is shown in the third column of this Table and it is computed as the difference between the mean error of all four melodies ($\mu$ value) in the initial and the last generations over the error ($\mu$ value) in the initial generation for all simulations. The relative improvement of the mean values in all simulations mostly are between 0.32 and 0.46, showing minor differences among different rater–setup combinations. The statistical significance of these differences is discussed after the following paragraph.

Additional exploration of the convergence speed is performed by measuring the rating iteration in which the 90 % of the total relative improvement is achieved. The selection of the 90 % limit is abstract, it could be any percentage that approaches 100 %. This measurement provides insights about the expected rating round (PSO iteration) in which the user will have covered most of the progress (i.e. 90 % of the system’s optimal fitness improvement capabilities) having considered a maximum iteration limit of 20 rating rounds. Thereafter, the progress of the melodies is expected to be slower, since a small part of the potential improvement will be realizable (e.g. 10 %). Hence, the 90 % progress iteration is used as a means to identify the rating round at which a satisfactory improvement in melodies (in accordance to the system’s capabilities) will have been performed. After the 90 % fitness progress rating round, the deceleration of fitness improvement will imminently give the user the essence that the system is not responding to the ratings and fatigue will imminently have more chances to begin.

**Table 1:** Statistics of mean fitness improvement among the melodies of the initial and the final rating iteration, for all raters and setups, in all the respective simulations

Rater–setup	Init. fit.	Last fit.	Rel. impr.	90 % iter.
$$\hbox {R}_1$$				
$$\hbox {S}_1$$				
Rhythm	10.19 (1.94)	6.61 (1.28)	0.35 (0.11)	5.92 (4.08)
Tone	6.14 (1.37)	3.93 (1.03)	0.36 (0.15)	4.58 (2.26)
$$\hbox {S}_2$$				
Rhythm	10.04 (1.80)	6.66 (1.36)	0.33 (0.12)	5.38 (2.70)
Tone	6.21 (1.61)	3.78 (1.05)	0.40 (0.16)	4.06 (1.96)
$$\hbox {S}_3$$				
Rhythm	10.03 (1.76)	6.41 (1.46)	0.36 (0.13)	6.00 (3.12)
Tone	6.18 (1.70)	3.68 (1.07)	0.40 (0.16)	5.52 (3.59)
$$\hbox {S}_4$$				
Rhythm	10.24 (1.94)	6.91 (1.45)	0.32 (0.13)	5.82 (3.15)
Tone	6.05 (1.47)	3.67 (0.87)	0.39 (0.14)	4.80 (2.21)
$$\hbox {R}_2$$				
$$\hbox {S}_1$$				
Rhythm	10.57 (1.80)	6.70 (1.73)	0.37 (0.14)	6.50 (3.14)
Tone	6.31 (1.78)	3.55 (0.97)	0.44 (0.15)	5.62 (3.22)
$$\hbox {S}_2$$				
Rhythm	10.28 (1.79)	6.40 (1.69)	0.38 (0.14)	6.06 (2.90)
Tone	6.20 (1.41)	3.88 (1.23)	0.38 (0.18)	4.02 (1.72)
$$\hbox {S}_3$$				
Rhythm	10.38 (1.94)	6.40 (1.60)	0.38 (0.11)	7.96 (4.63)
Tone	6.25 (1.70)	3.37 (1.07)	0.46 (0.17)	5.62 (3.19)
$$\hbox {S}_4$$				
Rhythm	10.10 (1.80)	6.46 (1.83)	0.36 (0.15)	7.40 (4.38)
Tone	6.37 (1.76)	3.77 (1.09)	0.41 (0.15)	6.80 (4.93)
$$\hbox {R}_3$$				
$$\hbox {S}_1$$				
Rhythm	10.29 (1.79)	6.08 (1.60)	0.41 (0.14)	6.92 (3.97)
Tone	6.16 (1.75)	3.52 (1.09)	0.42 (0.18)	4.98 (3.14)
$$\hbox {S}_2$$				
Rhythm	10.31 (1.82)	6.49 (1.58)	0.37 (0.11)	5.62 (2.22)
Tone	6.25 (1.66)	3.74 (1.19)	0.41 (0.16)	4.10 (1.49)
$$\hbox {S}_3$$				
Rhythm	10.44 (1.81)	6.46 (1.65)	0.38 (0.13)	5.66 (2.31)
Tone	6.32 (1.67)	3.91 (1.18)	0.38 (0.15)	10.38 (4.15)
$$\hbox {S}_4$$				
Rhythm	10.03 (1.64)	6.28 (1.52)	0.37 (0.13)	6.96 (4.34)
Tone	6.20 (1.70)	3.47 (1.05)	0.44 (0.16)	6.00 (3.98)
$$\hbox {R}_4$$				
$$\hbox {S}_1$$				
Rhythm	10.64 (1.68)	6.69 (1.68)	0.37 (0.15)	6.36 (2.89)
Tone	6.18 (1.36)	3.55 (1.00)	0.42 (0.16)	5.06 (2.13)
$$\hbox {S}_2$$				
Rhythm	10.28 (1.77)	6.87 (1.68)	0.33 (0.13)	5.42 (2.60)
Tone	6.13 (1.64)	3.92 (1.37)	0.37 (0.20)	4.18 (2.40)
$$\hbox {S}_3$$				
Rhythm	10.33 (1.84)	6.59 (1.69)	0.36 (0.13)	7.56 (4.58)
Tone	6.04 (1.52)	3.69 (1.05)	0.39 (0.16)	5.74 (4.12)
$$\hbox {S}_4$$				
Rhythm	10.21 (1.85)	6.21 (1.62)	0.39 (0.13)	6.66 (3.91)
Tone	6.16 (1.82)	3.48 (1.22)	0.44 (0.18)	5.54 (3.75)

The statistical significance in the mean relative improvements for all raters and PSO setups is demonstrated in Table 2, where a “+” sign denotes that the improvement that corresponds to the rater and setup of the row is significantly higher than the one of the respective column. A “−” denotes the opposite. An “=” sign is shown if there is no statistical significance in the considered relative improvements. Each rater incorporates an upper-diagonal quadruple of sign symbols. Each row within each rater’s quadruple denotes the respective PSO setup. Therefore, the diagonal quadruples refer to the results of the statistical significance tests among the measurements of different setups for the same automatic rater, while the off diagonal (or upper-diagonal) quadruples refer to the tests’ results regarding all different raters and all PSO setups.

The statistical significance is measured through a two-sided Wilcoxon (1945) rank sum test, which is applied on the distributions of the 50 simulations for each rater and setup combination. Through this test, the statistical significance of the difference in the distributions of the relative improvements of each rater–setup combination is examined. Specifically, for each pair of rater–setup relative improvement measurements we employ the rank sum test to each respective pair, to obtain the probability that these two measurement sets belong to a continuous distribution with equal medians. Formally, the null hypothesis for each pair of relative improvement measurements is that they are independent samples from identical continuous distributions with equal medians. If the null hypothesis is rejected at the 5 % significance level for a pair of rater–setup, then these improvements are indicated to be significantly different in a statistical sense.

The analysis of the statistical significance in the improvement differences allows to distinguish whether there are rating profiles or PSO parameters setups which allow the system to function more efficiently. Table 2 indicates that there is statistically significant difference between some rating profile and setup combinations. Regarding the rhythm PSO swarms, the relative improvement is significantly smaller for some setups of the $\hbox {R}_1$ rater, especially for the $\hbox {S}_4$ PSO parameters setup. The tone swarm incorporates less combinations of significant inequalities than the rhythm swarm for the $\hbox {R}_1$ rater, but also presents some instances of significant superiority of the $\hbox {R}_2$ rater over $\hbox {R}_3$. Consulting the “relative improvement” column in Table 1, it may be noticed that either the $\hbox {S}_3$ or the $\hbox {S}_4$ setups provides the best improvement for all raters except from the rhythm swarm of rater $\hbox {R}_3$. Furthermore, by conducting the Wilcoxon test over all setup pairs, including the mean relative improvements of all raters, there is no statistical superiority of any setup. Therefore, concerning the mean relative improvement from the initial to the last rating round among all four individuals, the *r*-PSO is statistically equivalent to the standard PSO.

**Table 2:** Statistical significance of the differences in relative improvements of the mean fitness of all four melodies throughout all rating rounds among all raters and simulations

	$\hbox {R}_1$				$\hbox {R}_2$				$\hbox {R}_3$				$\hbox {R}_4$			
	Rhythm															
$\hbox {R}_1$	$=$	$=$	$=$	$=$	$=$	$=$	$=$	$=$	−	$=$	$=$	$=$	$=$	$=$	$=$	$=$
		$=$	$=$	$=$	$=$	$=$	−	$=$	−	$=$	$=$	$=$	$=$	$=$	$=$	−
			$=$	$=$	$=$	$=$	$=$	$=$	$=$	$=$	$=$	$=$	$=$	$=$	$=$	$=$
				$=$	$=$	−	−	$=$	−	$=$	−	$=$	−	$=$	$=$	−
$\hbox {R}_2$					$=$	$=$	$=$	$=$	$=$	$=$	$=$	$=$	$=$	$=$	$=$	$=$
						$=$	$=$	$=$	$=$	$=$	$=$	$=$	$=$	$=$	$=$	$=$
							$=$	$=$	$=$	$=$	$=$	$=$	$=$	$=$	$=$	$=$
								$=$	$=$	$=$	$=$	$=$	$=$	$=$	$=$	$=$
$\hbox {R}_3$									$=$	$=$	$=$	$=$	$=$	$+$	$=$	$=$
										$=$	$=$	$=$	$=$	$=$	$=$	$=$
											$=$	$=$	$=$	$=$	$=$	$=$
												$=$	$=$	$=$	$=$	$=$
$\hbox {R}_4$													$=$	$=$	$=$	$=$
														$=$	$=$	−
															$=$	$=$
																$$=$$
	Tone															
$\hbox {R}_1$	$=$	$=$	$=$	$=$	−	$=$	−	$=$	$=$	$=$	$=$	−	−	$=$	$=$	−
		$=$	$=$	$=$	$=$	$=$	−	$=$	$=$	$=$	$=$	$=$	$=$	$=$	$=$	$=$
			$=$	$=$	$=$	$=$	$=$	$=$	$=$	$=$	$=$	$=$	$=$	$=$	$=$	$=$
				$=$	$=$	$=$	−	$=$	$=$	$=$	$=$	$=$	$=$	$=$	$=$	$=$
$\hbox {R}_2$					$=$	$=$	$=$	$=$	$=$	$=$	$+$	$=$	$=$	$=$	$=$	$=$
						$=$	−	$=$	$=$	$=$	$=$	$=$	$=$	$=$	$=$	$=$
							$=$	$=$	$=$	$=$	$+$	$=$	$=$	$+$	$+$	$=$
								$=$	$=$	$=$	$=$	$=$	$=$	$=$	$=$	$=$
$\hbox {R}_3$									$=$	$=$	$=$	$=$	$=$	$=$	$=$	$=$
										$=$	$=$	$=$	$=$	$=$	$=$	$=$
											$=$	−	$=$	$=$	$=$	$=$
												$=$	$=$	$=$	$=$	$=$
$\hbox {R}_4$													$=$	$=$	$=$	$=$
														$=$	$=$	$=$
															$=$	$=$
																$$=$$

A question that rise concerns the reason for the “inability” of the $\hbox {R}_1$ rater to produce relative improvements that are statistically comparable with the ones presented by the other raters, for some setups. The answer in this question lies within the rating values of $\hbox {R}_1$. A fundamental statistical analysis of the raters’ ratings is demonstrated in Table 3. The values presented therein concern the ratings in the initial and the final rating round (PSO iteration), as well as their absolute and relative differences. The second column of this Table reveals that the $\hbox {R}_1$ ratings nearly reached the maximum rate of 10. Consulting Fig. 4, it is noticed that the rating curve that corresponds to rater 1 ($\hbox {R}_1$) reaches a near-zero absolute gradient plateau when ratings (y-axis) exceed the level of 9. Therefore, the rating behavior of $\hbox {R}_1$ indicates that this automatic rater is almost completely satisfied by the output of the melodies at an “early” evolutionary stage and further improvement is not required, since all the presented melodies are rated almost equally high (near 10) after a rating round.

Moreover, by consulting the 90 % iteration column of Table 1, it is observed that $\hbox {R}_1$ provides rates near 9 at an early stage of the PSO evolution, i.e. from around 4 to 6 rounds for rhythms and tones. This is a clear indication that the maximum performance has been almost reached and no further improvements are necessary. Additionally, the reasons for the smaller improvement of the $\hbox {R}_1$ rater, as have hitherto been analyzed, are amplified by the randomness in the provided rates. Thereby, the additional noise in ratings makes alterations to the rate (fitness) of a PSO agent that are more decisive than actual fitness improvements. Having in mind the near-flat plateau around rate 9 for rater 1 in Fig. 4, it is clear that a small improvement in rates is realized through a large improvement in fitness. Under this perspective, the $\pm 0.5$ randomness margin (the $\xi _{\beta }$ value in Eq. 9) in ratings is considered as an extensive potential perturbation, which further obstructs the evolutionary process. It is thus deduced that the rating behavior of $\hbox {R}_1$ does not expose a weakness of the system, but a rating convergence to a “noisy global maximum”. This rating behavior may be interpreted as the behavior of a human rater who is completely satisfied, within the limits of aesthetic art uncertainty, by the system’s output even from an early rating round.

**Table 3:** Statistics of mean rating improvement from the initial to the final rating iterations, for all raters and setups, in all the respective simulations

Rater–setup	Init. rate	Last rate	Abs. impr.	Rel. impr.
$$\hbox {R}_1$$				
$$\hbox {S}_1$$				
Rhythm	5.63 (2.69)	9.20 (0.67)	3.57 (1.53)	0.93 (1.47)
Tone	5.88 (2.70)	9.11 (1.01)	3.29 (1.24)	0.63 (0.39)
$$\hbox {S}_2$$				
Rhythm	5.87 (2.55)	9.17 (0.68)	3.30 (1.44)	0.66 (0.45)
Tone	5.72 (2.97)	9.15 (1.14)	3.55 (1.28)	0.68 (0.37)
$$\hbox {S}_3$$				
Rhythm	5.95 (2.55)	9.25 (0.78)	3.30 (1.43)	0.64 (0.45)
Tone	5.84 (2.94)	9.23 (1.02)	3.39 (1.30)	0.63 (0.36)
$$\hbox {S}_4$$				
Rhythm	5.51 (2.77)	9.00 (1.05)	3.51 (1.65)	0.82 (0.74)
Tone	6.04 (2.81)	9.33 (0.69)	3.30 (1.11)	0.58 (0.27)
$$\hbox {R}_2$$				
$$\hbox {S}_1$$				
Rhythm	2.80 (1.79)	6.59 (1.59)	3.78 (1.37)	1.56 (0.92)
Tone	3.31 (2.23)	7.25 (1.38)	4.00 (1.40)	1.34 (0.70)
$$\hbox {S}_2$$				
Rhythm	3.10 (1.78)	6.82 (1.51)	3.72 (1.36)	1.43 (0.85)
Tone	3.32 (2.00)	6.76 (1.80)	3.55 (1.60)	1.16 (0.79)
$$\hbox {S}_3$$				
Rhythm	3.02 (1.93)	6.86 (1.48)	3.84 (1.15)	1.63 (1.28)
Tone	3.32 (2.14)	7.49 (1.49)	4.17 (1.51)	1.36 (0.81)
$$\hbox {S}_4$$				
Rhythm	3.28 (1.89)	6.79 (1.65)	3.51 (1.41)	1.29 (0.95)
Tone	3.21 (2.12)	6.91 (1.56)	3.81 (1.35)	1.31 (0.70)
$$\hbox {R}_3$$				
$$\hbox {S}_1$$				
Rhythm	1.86 (1.21)	5.11 (1.44)	3.25 (1.25)	1.93 (0.94)
Tone	2.18 (1.52)	5.17 (1.45)	3.01 (1.47)	1.57 (1.06)
$$\hbox {S}_2$$				
Rhythm	1.88 (1.22)	4.71 (1.37)	2.83 (1.06)	1.84 (1.36)
Tone	2.07 (1.44)	4.95 (1.60)	2.93 (1.38)	1.56 (1.04)
$$\hbox {S}_3$$				
Rhythm	1.78 (1.19)	4.76 (1.40)	2.98 (1.13)	1.86 (1.09)
Tone	1.98 (1.47)	4.67 (1.52)	2.69 (1.21)	1.47 (0.88)
$$\hbox {S}_4$$				
Rhythm	2.02 (1.18)	4.88 (1.32)	2.87 (1.19)	1.73 (1.48)
Tone	2.16 (1.48)	5.24 (1.43)	3.12 (1.34)	1.58 (0.89)
$$\hbox {R}_4$$				
$$\hbox {S}_1$$				
Rhythm	0.78 (0.54)	2.36 (0.93)	1.59 (0.82)	2.40 (1.81)
Tone	0.99 (0.65)	2.71 (0.93)	1.74 (0.84)	1.94 (1.18)
$$\hbox {S}_2$$				
Rhythm	1.87 (1.20)	4.44 (1.41)	2.59 (1.13)	1.67 (1.23)
Tone	2.18 (1.39)	4.69 (1.85)	2.64 (1.62)	1.31 (0.93)
$$\hbox {S}_3$$				
Rhythm	1.87 (1.24)	4.69 (1.46)	2.83 (1.10)	1.70 (0.89)
Tone	2.25 (1.53)	4.96 (1.46)	2.73 (1.27)	1.30 (0.73)
$$\hbox {S}_4$$				
Rhythm	1.95 (1.20)	4.97 (1.42)	3.02 (1.24)	1.85 (1.40)
Tone	2.24 (1.49)	5.28 (1.63)	3.11 (1.45)	1.52 (0.91)

**Fig. 6:**
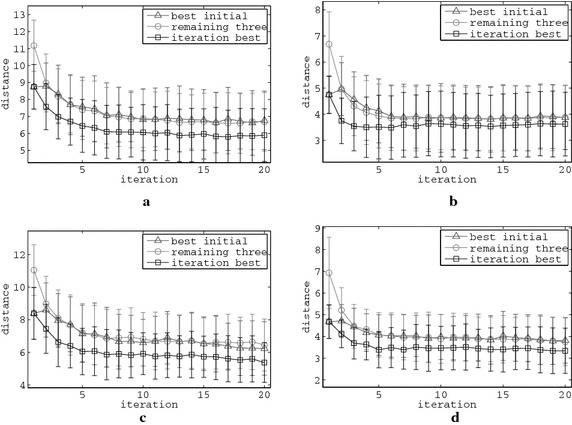
Errorbars of selected agents’ fitness in all 50 simulations of representative PSO setups for all automatic raters. Specifically, errorbars of the fitness are demonstrated for all 20 rating iterations of the agent with the best fitness in the initial iteration, for the remaining three agents and the for the best fit agent in all iterations.** a** $\hbox {R}_2$-$\hbox {S}_1$ rhythm.** b** $\hbox {R}_2$-$\hbox {S}_2$ tone. **c** $\hbox {R}_2$-$\hbox {S}_3$ rhythm.** d** $\hbox {R}_2$-$\hbox {S}_4$ tone

Through the initialisation process four initial melodies are generated that are represented by four points in the rhythm and tone spaces that are possibly separated by different distances from the point that represents the ideal features, both in the rhythm and the tone feature spaces. Therefore, the fitness of one among the initial agents is expected to be better than the fitness of the others, i.e. the fitness of the agent that produced a melody, the features of which are placed closer to the ideal features. Figure 6 provides a statistical graphical answer for some rater–setup examples to the following question: “is the initially best fit agent the one that remains best fit throughout all 20 rating rounds?” This figure suggests a negative answer, meaning that the best fit agent in each iteration is expected be different than the one of the initial iteration. Additionally, it may also be observed that the remaining three agents, excluding the best fit in each iteration, gradually generate melodies, the features of which are closer to the ideal features. This fact amplifies the indications yielded so far that the melodies which represent all swarm members “converge” to the ideal melody that each rater may anticipate. The respective graphs of the remaining rater–setup combinations exhibit a similar behaviour.

Table 4 presents the basic statistics of the fitness values among the *best* fit melodies in the initial and the last rating iteration. The findings in this Table, when combined with the respective mean fitness values of all four melodies that comprise the initial and the last iterations in Table 1, allow a numeric estimation of the fitness convergence behaviour of the system. By comparing Tables 4 and 1 it is observed that the relative improvement of the agents’ mean fitness is similar to the one of the best agent’s at each iteration. This is also graphically observed by the coordinated fitness reduction of the “iteration best” and “remaining three” curves in the examples depicted in Fig. 6. Considering also the fact that the fitness of the best melody remains significantly better than of the rest melodies even at the last generation step, it is implied that a distinguishably more “pleasant” melody is expected to be present throughout the entire rating procedure.

**Table 4:** Statistics of the best melody’s fitness improvement from the initial to the final rating iteration, for all raters and setups, in all the respective simulations

Rater–setup	Best init. fit.	Best last fit.	Rel. impr.	$90\,\%$ iter.
$$\hbox {R}_1$$				
$$\hbox {S}_1$$				
Rhythm	8.26 (1.76)	4.98 (1.27)	0.39 (0.15)	8.62 (4.79)
Tone	4.74 (0.77)	2.89 (0.74)	0.38 (0.17)	6.28 (4.79)
$$\hbox {S}_2$$				
Rhythm	8.12 (1.35)	5.22 (1.31)	0.35 (0.15)	6.12 (3.63)
Tone	4.54 (0.76)	2.88 (0.82)	0.36 (0.19)	3.98 (3.63)
$$\hbox {S}_3$$				
Rhythm	8.07 (1.30)	4.45 (0.94)	0.44 (0.12)	9.22 (4.41)
Tone	4.55 (0.89)	2.49 (0.77)	0.44 (0.19)	7.94 (4.41)
$$\hbox {S}_4$$				
Rhythm	8.30 (1.58)	5.00 (1.09)	0.38 (0.15)	9.54 (4.31)
Tone	4.45 (0.84)	2.51 (0.57)	0.42 (0.16)	7.64 (4.31)
$$\hbox {R}_2$$				
$$\hbox {S}_1$$				
Rhythm	8.75 (1.32)	5.27 (1.47)	0.40 (0.14)	9.10 (4.24)
Tone	4.57 (0.98)	2.63 (0.67)	0.41 (0.17)	7.04 (4.24)
$$\hbox {S}_2$$				
Rhythm	8.57 (1.36)	5.15 (1.50)	0.40 (0.16)	6.52 (4.26)
Tone	4.75 (0.72)	2.91 (0.88)	0.37 (0.21)	5.12 (4.26)
$$\hbox {S}_3$$				
Rhythm	8.39 (1.59)	4.72 (1.23)	0.43 (0.16)	10.76 (5.48)
Tone	4.49 (0.85)	2.31 (0.79)	0.47 (0.19)	8.92 (5.48)
$$\hbox {S}_4$$				
Rhythm	8.31 (1.47)	4.76 (1.54)	0.42 (0.15)	10.14 (4.88)
Tone	4.68 (0.77)	2.72 (0.64)	0.40 (0.17)	8.84 (4.88)
$$\hbox {R}_3$$				
$$\hbox {S}_1$$				
Rhythm	8.34 (1.26)	4.90 (1.42)	0.41 (0.15)	8.32 (4.66)
Tone	4.50 (0.96)	2.59 (0.73)	0.40 (0.20)	7.04 (4.66)
$$\hbox {S}_2$$				
Rhythm	8.52 (1.39)	5.21 (1.23)	0.39 (0.11)	6.96 (4.44)
Tone	4.61 (0.77)	2.79 (0.83)	0.39 (0.19)	5.80 (4.44)
$$\hbox {S}_3$$				
Rhythm	8.43 (1.48)	4.66 (1.32)	0.44 (0.15)	8.68 (4.52)
Tone	4.61 (0.86)	2.65 (0.68)	0.41 (0.16)	10.92 (4.52)
$$\hbox {S}_4$$				
Rhythm	8.42 (1.30)	4.59 (1.03)	0.45 (0.14)	8.96 (4.90)
Tone	4.52 (0.95)	2.40 (0.68)	0.44 (0.20)	8.38 (4.90)
$$\hbox {R}_4$$				
$$\hbox {S}_1$$				
Rhythm	8.82 (1.17)	5.41 (1.39)	0.39 (0.14)	8.18 (4.13)
Tone	4.81 (0.71)	2.73 (0.76)	0.42 (0.19)	6.18 (4.13)
$$\hbox {S}_2$$				
Rhythm	8.42 (1.45)	5.63 (1.52)	0.32 (0.17)	7.72 (4.93)
Tone	4.59 (0.73)	2.97 (0.88)	0.34 (0.20)	4.54 (4.93)
$$\hbox {S}_3$$				
Rhythm	8.38 (1.37)	4.99 (1.35)	0.40 (0.15)	11.40 (5.37)
Tone	4.48 (0.99)	2.63 (0.82)	0.39 (0.20)	7.50 (5.37)
$$\hbox {S}_4$$				
Rhythm	8.30 (1.25)	4.49 (1.05)	0.45 (0.14)	9.36 (4.98)
Tone	4.45 (0.81)	2.48 (0.79)	0.43 (0.20)	8.26 (4.98)

Regarding the relative improvements of the best fit melodies between the initial and the final rating iteration, the results are similar to the ones presented for the mean fitness of all agents presented earlier. Table 5 demonstrates that there are many statistically significant instances of $\hbox {S}_2$ inferiority for the rhythm of the $\hbox {R}_4$ rater. Additionally, the $\hbox {S}_3$ setup in the tone swarm of $\hbox {R}_2$, is significantly superior over many other rater–setup combinations. Nevertheless, the overall impression is that there are generally no statistically significant differences between rater–setup pairs, indicating that the *r*-PSO modification is not inferior to the standard PSO methodology. Moreover, although mostly not statistically significant, the setups that utilize the *r*-PSO variation achieve the best performance towards improving the best rated individual, as demonstrated in the “relative improvement” column of Table 4.

**Table 5:** Statistical significance of the differences in relative improvements of the best melodies throughout all rating rounds, among all raters and simulations

	$\hbox {R}_1$				$\hbox {R}_2$				$\hbox {R}_3$				$\hbox {R}_4$			
	Rhythm															
$\hbox {R}_1$	$=$	$=$	−	$=$	$=$	$=$	$=$	$=$	$=$	$=$	$=$	−	$=$	$+$	$=$	$=$
		$=$	−	$=$	$=$	$=$	−	−	$=$	$=$	−	−	$=$	$=$	$=$	−
			$=$	$+$	$=$	$=$	$=$	$=$	$=$	$+$	$=$	$=$	$=$	$+$	$=$	$=$
				$=$	$=$	$=$	$=$	$=$	$=$	$=$	−	−	$=$	$=$	$=$	−
$\hbox {R}_2$					$=$	$=$	$=$	$=$	$=$	$=$	$=$	−	$=$	$+$	$=$	$=$
						$=$	$=$	$=$	$=$	$=$	$=$	$=$	$=$	$+$	$=$	$=$
							$=$	$=$	$=$	$=$	$=$	$=$	$=$	$+$	$=$	$=$
								$=$	$=$	$=$	$=$	$=$	$=$	$+$	$=$	$=$
$\hbox {R}_3$									$=$	$=$	$=$	$=$	$=$	$+$	$=$	$=$
										$=$	−	−	$=$	$=$	$=$	−
											$=$	$=$	$=$	$+$	$=$	$=$
												$=$	$+$	$+$	$=$	$=$
$\hbox {R}_4$													$=$	$=$	$=$	$=$
														$=$	−	−
															$=$	$=$
																$$=$$
	Tone															
$\hbox {R}_1$	$=$	$=$	$=$	$=$	$=$	$=$	−	$=$	$=$	$=$	$=$	−	$=$	$=$	$=$	$=$
		$=$	−	$=$	$=$	$=$	−	$=$	$=$	$=$	$=$	−	$=$	$=$	$=$	$=$
			$=$	$=$	$=$	$=$	$=$	$=$	$=$	$=$	$=$	$=$	$=$	$+$	$=$	$=$
				$=$	$=$	$=$	$=$	$=$	$=$	$=$	$=$	$=$	$=$	$+$	$=$	$=$
$\hbox {R}_2$					$=$	$=$	−	$=$	$=$	$=$	$=$	$=$	$=$	$=$	$=$	$=$
						$=$	−	$=$	$=$	$=$	$=$	$=$	$=$	$=$	$=$	$=$
							$=$	$+$	$=$	$+$	$+$	$=$	$=$	$+$	$+$	$=$
								$=$	$=$	$=$	$=$	$=$	$=$	$=$	$=$	$=$
$\hbox {R}_3$									$=$	$=$	$=$	$=$	$=$	$=$	$=$	$=$
										$=$	$=$	$=$	$=$	$=$	$=$	$=$
											$=$	$=$	$=$	$=$	$=$	$=$
												$=$	$=$	$+$	$=$	$=$
$\hbox {R}_4$													$=$	$+$	$=$	$=$
														$=$	$=$	−
															$=$	$=$
																$$=$$

### Music features convergence analysis

The rating process incorporates the rating of four melodies at each round (PSO iteration). The analysis that has hitherto been performed, incorporates the improvement in fitness and ratings of the music composed according to the features that are encompassed to the rhythm and tone PSO agents. The perspective of these results does not only revolve around the effectiveness of the system, but also on the anticipated user fatigue imposed by the converging behaviour. However, the convergence analysis of the system so far, considered only the fitness aspects and not the melodic characteristics. A human user is expected to be affected by the differences in the music content of the four melodies that comprise the rating round. Specifically, if the system presents four melodies that exhibit similar characteristics from an early rating round, then the user may lose focus by considering that the system has more or less evolved the melody as far as it could. Therefore, it is important to examine the evolution of melodic “distances” among the melodies both throughout and within each rating round. The melodic distances are expressed through distances in the music feature (rhythmic and tonal) that are extracted from the melodies ($\vec {f_{c,i}^{r}}$ and $\vec {f_{c,i}^{t}}$) composed under the respective agents’ guidelines ($\vec {f_{a,i}^{r}}$ and $\vec {f_{a,i}^{t}}$, $i \in \{ 1,2,3,4 \}$).

To this end, the aforementioned musical convergence analysis is performed by analysing the location difference of agents’ melodies both in successive iterations and the inter-agent distances within single rating rounds. Hereafter, the music features of the compositions in the *k*-th iteration ($k = 1,2,\dots ,20$) will be denoted as $\vec {f_{c,i}^{x}}(k)$, where $i \in \{ 1,2,3,4 \}$ and $x \in \{ r,t \}$. The melodic distance between the successive locations of an agent’s melody in the feature space is a vector with 19 elements, considering a total number of 20 iterations. Using the above mentioned denotations, the successive distances vector of the *i*-th agent is computed by

11 $$\vec {d_{i}^{x}}(m) = \left\| \vec {f_{c,i}^{x}}(m+1) - \vec {f_{c,i}^{x}}(m) \right\| _2 ,$$

where $m \in \{ 1,2,\dots ,19 \}$ and $x \in {r,t,\text {all}}$. This examination incorporates not only the isolated rhythm and tone feature vectors, but also the overall music distance described by the vector comprising all 39 features (22 rhythmic and 17 tonal). The merged successive distance vector is incorporated in the $\vec {d_{i}^{\mathrm {all}}}$ vector. It is again noticed that each distance vector comprises successive distances between the features of the melodies that were composed under the guidance of the respective agents and not the features of the agents per se. This examination concerns the differences in the music output throughout the rating iterations and not the “orbits” of the PSO agents.

**Fig. 7:**
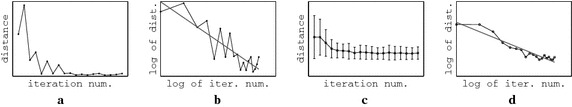
**a** Distances between successive steps (from iteration $i-1$ to *i*) of an agent. **b** The log–log plot of the the distances in (**a**) and the optimally fit line in a least squares sense. **c** *Error bars* of the distances in all agent’s successive steps. **d** Log–log of the mean values in (**c**) and the optimally fit line in a least squares sense

A graphical example of a successive distances vector is depicted in Fig. 7a, where the distance values are exhibited to decrease in a pattern that resembles a pow law. In Fig. 7b, the aforementioned distance vector is plotted on a log–log scale and a regression line is optimally fitted, in a least squares sense, through its point. The gradient of this line indicates the rate that successive melodic distances decrease, with larger absolute values signifying a faster successive distances reduction. Figure 7c and d demonstrate that the mean distance reduction of all agents approaches a power low pattern. Figure 7c illustrates the error bars of the successive distances vectors of all the 200 melodies composed in 50 simulations of a representative rater–setup pair. The log–log plot and the regression line of the mean values in Figure 7c are illustrated in Figure 7d.

Table 6 demonstrates the gradients of the regression lines through the vector of successive distances, as computed in Eq. 11, in the log–log scale (as appeared in Fig. 7b) for all rater–setup combinations. By examining the “per $\hbox {S}_i$” rows of the above mentioned table, which exhibits the mean gradient per setup, it is evident that there are major differences regarding the reduction in the successive distances for the agents orbits per PSO parameter setup, for the rhythm, tone and the merged features. Additionally, almost all gradient distributions for every pair of setups are statistically significantly different, again for all three examined vectors. The differences in the statistical significance are shown by the “$+-=$” signs on the exponents of each rater and setup. The distance reduction within all the measurements of the respective rater or setups are signified by a “$+$”, a “−” or a “$=$” sign, if the rater or setup under discussion exhibits greater, smaller or statistically equal gradient. For example, the rhythm distributions of setup $\hbox {S}_{1}^{=+{-}{-}}$ demonstrates significantly larger gradients compared to $\hbox {S}_{2}$ and smaller compared to $\hbox {S}_{3}$ and $\hbox {S}_{4}$. Contrarily to the setups, there are little statistically significant difference among the gradients’ distributions for any pair of raters and for any distance vector, for the measurements that are demonstrated in the “per $\hbox {R}_i$” column.

**Table 6:** Distance reduction in successive rhythm, tone and overall features throughout all rating steps

	$\hbox {S}_{1}^{=+{-}{-}}$	$\hbox {S}_{2}^{-={-}{-}}$	$\hbox {S}_{3}^{++==}$	$\hbox {S}_{4}^{++==}$	per $\hbox {R}_i$
	Rhythm ($\vec {d_{i}^{r}}$ reduction)				
$\hbox {R}_{1}^{====}$	−0.52 (0.61)	−0.69 (0.59)	−0.14 (0.45)	−0.15 (0.44)	−0.37 (0.58)
$\hbox {R}_{2}^{====}$	−0.54 (0.58)	−0.71 (0.63)	−0.22 (0.45)	−0.23 (0.47)	−0.43 (0.58)
$\hbox {R}_{3}^{====}$	−0.56 (0.64)	−0.64 (0.54)	−0.22 (0.36)	−0.24 (0.39)	−0.42 (0.53)
$\hbox {R}_{4}^{====}$	−0.55 (0.53)	−0.67 (0.58)	−0.21 (0.44)	−0.24 (0.44)	−0.42 (0.54)
per $\hbox {S}_i$	−0.54 (0.59)	−0.68 (0.58)	−0.20 (0.42)	−0.22 (0.44)	
	Tone ($\vec {d_{i}^{t}}$ reduction)				
$\hbox {R}_{1}^{=+==}$	−0.54 (0.33)	−0.64 (0.33)	−0.26 (0.28)	−0.32 (0.27)	−0.44 (0.34)
$\hbox {R}_{2}^{-==-}$	−0.57 (0.31)	−0.63 (0.33)	−0.35 (0.27)	−0.33 (0.31)	−0.47 (0.33)
$\hbox {R}_{3}^{====}$	−0.52 (0.31)	−0.63 (0.30)	−0.33 (0.27)	−0.33 (0.25)	−0.45 (0.31)
$\hbox {R}_{4}^{=+==}$	−0.55 (0.32)	−0.55 (0.34)	−0.28 (0.27)	−0.33 (0.27)	−0.43 (0.33)
per $\hbox {S}_i$	−0.55 (0.32)	−0.61 (0.33)	−0.30 (0.27)	−0.32 (0.28)	
	Rhythm and tone ($\vec {d_{i}^{\mathrm {all}}}$ reduction)				
$\hbox {R}_{1}^{=+==}$	−0.54 (0.52)	−0.70 (0.52)	−0.16 (0.41)	−0.18 (0.38)	−0.39 (0.52)
$\hbox {R}_{2}^{-===}$	−0.57 (0.52)	−0.73 (0.54)	−0.25 (0.40)	−0.25 (0.43)	−0.45 (0.52)
$\hbox {R}_{3}^{====}$	−0.58 (0.53)	−0.65 (0.45)	−0.23 (0.34)	−0.25 (0.36)	−0.43 (0.47)
$\hbox {R}_{4}^{====}$	−0.57 (0.48)	−0.67 (0.49)	−0.23 (0.39)	−0.26 (0.40)	−0.43 (0.48)
per $\hbox {S}_i$	−0.56 (0.51)	−0.69 (0.50)	−0.22 (0.39)	−0.24 (0.39)	

Regarding the setups, the *r*-PSO variation produces significantly larger (smaller in an absolute value) gradients, revealing that each agent roams the feature space with bigger strides, producing melodies that are potentially more diverse. Thereby, the diversity in the melodic content provides the user with an impression that the system is able to compose diverse melodies. At the same time, these melodies converge to the ideal features as the rating iterations proceed, as indicated by the fact that all setups reached comparable fitness improvements as exhibited in Tables 1 and 4, with minor statistically significant superiorities between rater–setup pairs. Hence, it is expected that the robustness of the system will be preserved among the aforementioned examined setup versions, with different characteristics of the “melodic orbits”. Furthermore, the fact that the difference in distance reductions between different raters is generally not statistically significant is also encouraging, since the system’s melodic convergence behaviour is anticipated to remain unchanged when used by human users with different rating profiles.

Besides the inter-iteration distances of a single melody, it is also important to examine the melodic distances between the melodies that comprise each rating round. As also mentioned earlier in “Fitness convergence analysis” section, the initialisation scheme produces distant quadruples of agents and therefore the melodies composed under their guidelines are also expected to be distant. Since the fitness of all the agents is improving throughout the rating iteration, the melodies are contracting within an area of the feature space that is close to the rater’s ideal features. The question that raises concerns the speed of this melodic contraction, which may be described by the sum of distances between every agent pair, among the four ones in each iteration. A larger sum of inter-agent distances denotes the inclusion of a greater musical variety within the quadruple of the current rating iteration, while a smaller sum indicates a quadruple of similar melodies. Formally, the sum of inter-agent distances for each simulation is a 20-value vector, with each value describing the sum of each agents’ pairs distances per rating round, and is expressed as

12 $$\vec {\delta ^{x}}(k) = \sum _{i=1}^{4} \left( \sum _{j=i+1}^{4} \left\| \vec {f_{c,i}^{x}}(k) - \vec {f_{c,j}^{x}}(k) \right\| _2 \right) ,$$

where $k \in \{ 1,2,\dots ,20 \}$ denotes the rating iteration and $x \in \{ r,t,\text {all} \}$ again refers to the rhythmic, tonal and overall features respectively.

**Fig. 8:**
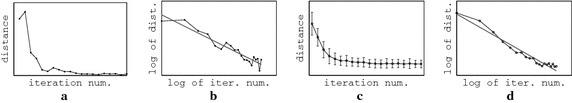
**a** Distances between every pair of agents in each iteration. **b** The log–log plot of the the distances in (**a**) and the optimally fit line in a least squares sense. **c** *Error bars* of the distances between all agents in all steps. **d** Log–log of the mean values in (**c**) and the optimally fit line in a least squares sense

Example illustrations of a simulation’s $\vec {\delta }$ vector are demonstrated in Fig. 8a, along with its log–log plot and the least squares regression line in Fig. 8b. Figure 8c depict the errorbars of the $\vec {\delta }$ vectors produced by all 50 simulation of a representative rater–setup pair, where it is observed that the mean values also resemble a power law reduction rate. Figure 8d illustrates the log–log plot and the regression line of the mean values in Fig. 8c. The melodic contraction is again measured through the gradient of the regression line, with larger absolute gradient values denoting a greater contraction rate, on contrary to smaller ones.

The melodic contraction gradients, as computed by Eq. 12, are demonstrated in Table 7 for rhythm, tone and the merged features. Again, the statistical significance of the difference in distributions among the contraction gradients of raters and setups are signified by the signs on the exponent. The inter-rater measurements do not exhibit statistically significant differences in the distributions of gradients, except from the $\hbox {R}_2$–$\hbox {R}_4$ rater pair. The distributions of the inter-setup distances present a similar behaviour to the ones examined in the previous paragraph. Again, the melodic contraction of the quadruples with the *r*-PSO variation is slower, providing the user with a more diverse collection of melodies in each rating round.

**Table 7:** Distance reduction between the features of all pairs of melodies in rating step

	Rhythm ($\vec {\delta ^{r}}$ reduction)				per $\hbox {R}_i$
	$\hbox {S}_{1}^{=+{-}{-}}$	$\hbox {S}_{2}^{-={-}{-}}$	$\hbox {S}_{3}^{++==}$	$\hbox {S}_{4}^{++==}$	
$\hbox {R}_{1}^{====}$	−0.75 (0.34)	−1.05 (0.43)	−0.60 (0.20)	−0.60 (0.22)	−0.75 (0.36)
$\hbox {R}_{2}^{====}$	−0.79 (0.35)	−1.13 (0.50)	−0.62 (0.24)	−0.62 (0.26)	−0.79 (0.41)
$\hbox {R}_{3}^{====}$	−0.84 (0.43)	−1.08 (0.44)	−0.59 (0.20)	−0.64 (0.22)	−0.79 (0.39)
$\hbox {R}_{4}^{====}$	−0.76 (0.30)	−0.99 (0.46)	−0.60 (0.26)	−0.62 (0.25)	−0.74 (0.36)
per $\hbox {S}_i$	−0.78 (0.35)	−1.06 (0.46)	−0.60 (0.23)	−0.62 (0.24)	

	Tone ($\vec {\delta ^{t}}$ reduction)				per $\hbox {R}_i$
	$\hbox {S}_{1}^{=+{-}{-}}$	$\hbox {S}_{2}^{-={-}{-}}$	$\hbox {S}_{3}^{++=+}$	$\hbox {S}_{4}^{++-=}$	
$\hbox {R}_{1}^{====}$	−0.78 (0.24)	−0.97 (0.28)	−0.68 (0.15)	−0.74 (0.23)	−0.79 (0.25)
$\hbox {R}_{2}^{===-}$	−0.84 (0.25)	−0.97 (0.27)	−0.68 (0.19)	−0.76 (0.23)	−0.81 (0.26)
$\hbox {R}_{3}^{====}$	−0.77 (0.27)	−1.00 (0.29)	−0.69 (0.18)	−0.69 (0.18)	−0.79 (0.26)
$\hbox {R}_{4}^{=+==}$	−0.82 (0.21)	−0.90 (0.25)	−0.61 (0.20)	−0.69 (0.19)	−0.76 (0.24)
per $\hbox {S}_i$	−0.81 (0.24)	−0.96 (0.27)	−0.67 (0.18)	−0.72 (0.21)	

	Rhythm and tone ($\vec {\delta ^{\mathrm {all}}}$ reduction)				per $\hbox {R}_i$
	$\hbox {S}_{1}^{=+{-}{-}}$	$\hbox {S}_{2}^{-={-}{-}}$	$\hbox {S}_{3}^{++==}$	$\hbox {S}_{4}^{++==}$	
$\hbox {R}_{1}^{====}$	−0.74 (0.33)	−1.03 (0.41)	−0.59 (0.20)	−0.60 (0.22)	−0.74 (0.35)
$\hbox {R}_{3}^{====}$	−0.78 (0.34)	−1.10 (0.45)	−0.62 (0.23)	−0.61 (0.26)	−0.78 (0.38)
$\hbox {R}_{3}^{====}$	−0.81 (0.39)	−1.06 (0.40)	−0.59 (0.19)	−0.64 (0.22)	−0.77 (0.36)
$\hbox {R}_{4}^{====}$	−0.76 (0.29)	−0.96 (0.42)	−0.59 (0.26)	−0.61 (0.24)	−0.73 (0.34)
per $\hbox {S}_i$	−0.77 (0.34)	−1.04 (0.42)	−0.60 (0.22)	−0.62 (0.23)	

### Adaptation of specific music features

The analysis so far focused on several aspects of fitness and rating improvement of the melodies, the musical “orbit” that each melody circumscribes throughout the rating rounds and the relations between the melodies that comprise each rating round from the beginning to the end of each simulation. The remaining of this section examines the fitness in the specific submodules that are included in the bottom level of the system, namely the binary rhythm, the polyphony, the intensity, the pause submodules and the tonal module. Furthermore, the analysis goes deeper to the fitness adaptation of the system on each specific feature that formulates the fitness criteria of the system’s respective comprising submodules. Additionally, the relations between ideal-to-melody ($\vec {f_{*}^{x}}$-to-$\vec {f_{c,i}^{x}}$) and agent-to-melody ($\vec {f_{a,i}^{x}}$-to-$\vec {f_{c,i}^{x}}$) features are scrutinised, in order to approach the extent at which the bottom level music composition submodules affect the systems performance and the PSO adaptation. It is reminded that hitherto only the ideal-to-melody distances have been examined, which constitutes the criterion for the system’s “musical convergence”.

Table 8 exhibits the mean distance per feature for each submodule between the ideal features ($\vec {f_{*}^{x}}$) and the features of the four melodies ($\vec {f_{c,i}^{x}}$) composed during the last rating iteration. This distance is computed as the mean value of the errors comprise the fitness criterion for each music composition submodule. For example, the binary selector submodule composes binary rhythm in accordance to the fitness provided by five features, the features numbered from 1 to 5 on the rhythm feature vector. The mean distance per feature of this submodule, between a melody and the target features is computed as the mean distance value only of these five aforementioned features. The same holds for the mean distances per feature between the features that are carried by the four agents ($\vec {f_{a,i}^{x}}$) and the ones that are encompassed into the four respectively composed melodies ($\vec {f_{c,i}^{x}}$). The latter distances are demonstrated in Table 9.

**Table 8:** Mean distance per feature of each submodule between the ideal features ($\vec {f_{*}^{x}}$) and the features of the composed melodies ($\vec {f_{c,i}^{x}}$) of the four melodies that comprise the final rating round within each simulation for all raters and setups

	$\hbox {S}_1$	$\hbox {S}_2$	$\hbox {S}_3$	$\hbox {S}_4$
$$\hbox {R}_1$$				
Binary	0.29 (0.23)	0.29 (0.24)	0.25 (0.21)	0.29 (0.23)
Polyphony	0.59 (0.33)	0.59 (0.33)	0.61 (0.32)	0.57 (0.33)
Intensity	0.63 (0.37)	0.66 (0.36)	0.63 (0.35)	0.64 (0.36)
Pause	0.45 (0.30)	0.42 (0.29)	0.44 (0.28)	0.42 (0.28)
Rhythm	0.50 (0.34)	0.50 (0.34)	0.50 (0.33)	0.49 (0.34)
Tone	0.45 (0.35)	0.45 (0.36)	0.46 (0.35)	0.45 (0.35)
$$\hbox {R}_2$$				
Binary	0.28 (0.25)	0.28 (0.25)	0.31 (0.27)	0.28 (0.24)
Polyphony	0.59 (0.35)	0.57 (0.34)	0.58 (0.33)	0.56 (0.35)
Intensity	0.63 (0.36)	0.63 (0.36)	0.64 (0.35)	0.64 (0.36)
Pause	0.44 (0.29)	0.39 (0.28)	0.44 (0.29)	0.43 (0.28)
Rhythm	0.50 (0.35)	0.48 (0.34)	0.50 (0.34)	0.49 (0.34)
Tone	0.44 (0.36)	0.43 (0.35)	0.44 (0.36)	0.42 (0.36)
$$\hbox {R}_1$$				
Binary	0.28 (0.23)	0.29 (0.25)	0.28 (0.25)	0.27 (0.24)
Polyphony	0.58 (0.34)	0.57 (0.34)	0.59 (0.33)	0.59 (0.33)
Intensity	0.63 (0.38)	0.63 (0.36)	0.62 (0.36)	0.63 (0.38)
Pause	0.43 (0.30)	0.44 (0.30)	0.45 (0.29)	0.43 (0.29)
Rhythm	0.49 (0.35)	0.50 (0.34)	0.50 (0.34)	0.49 (0.35)
Tone	0.44 (0.36)	0.45 (0.36)	0.44 (0.36)	0.43 (0.35)
$$\hbox {R}_1$$				
Binary	0.31 (0.26)	0.33 (0.28)	0.30 (0.25)	0.29 (0.25)
Polyphony	0.59 (0.33)	0.63 (0.33)	0.56 (0.33)	0.54 (0.33)
Intensity	0.63 (0.37)	0.67 (0.38)	0.63 (0.37)	0.62 (0.37)
Pause	0.44 (0.30)	0.48 (0.29)	0.44 (0.28)	0.41 (0.29)
Rhythm	0.50 (0.34)	0.54 (0.35)	0.49 (0.34)	0.48 (0.34)
Tone	0.43 (0.35)	0.43 (0.36)	0.44 (0.36)	0.44 (0.36)

**Fig. 9:**
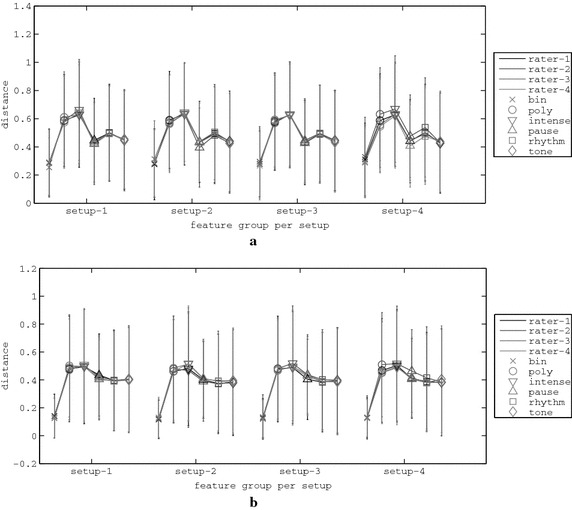
Errorbars of mean distances per feature of each submodule between target and composition features (**a**) and agent and composition features (**b**) for all raters and setups. **a** Distance between ideal and composition features. **b** Distance between agent and composition features

The findings in Tables 8 and 9 are also demonstrated graphically in Figs. 9a and b respectively, a fact that facilitates their interpretation. Specifically, it is clearly observable that the error distributions among all the music generation modules are highly related for all rater–setups combinations, in both graphs. An exact analysis on the relations and differences of these distributions may hardly contribute any vital information about the system’s performance, therefore it is omitted. It can be assumed, however, that the overall errors of the system (Table 8) are reflections of the errors produced by the underlying music composition modules (Table 9). In the aforementioned figure and tables, it is obvious that the binary selector produces the smallest errors among the other composition modules. Furthermore, the integer rhythm modules that define polyphony and intensity, exhibit a statistically higher error than the others.

**Table 9:** Mean distance per feature of each submodule between the features of the composed melodies ($\vec {f_{c,i}^{x}}$) and the features dictated by the respective agent ($\vec {f_{a,i}^{x}}$) of the four melodies that comprise the final rating round within each simulation for all raters and setups

	$\hbox {S}_1$	$\hbox {S}_2$	$\hbox {S}_3$	$\hbox {S}_4$
$$\hbox {R}_1$$				
Binary	0.14 (0.16)	0.14 (0.16)	0.13 (0.14)	0.13 (0.14)
Polyphony	0.48 (0.38)	0.49 (0.37)	0.50 (0.37)	0.47 (0.37)
Intensity	0.50 (0.41)	0.50 (0.41)	0.50 (0.41)	0.51 (0.40)
Pause	0.43 (0.29)	0.42 (0.31)	0.40 (0.28)	0.41 (0.29)
Rhythm	0.40 (0.36)	0.40 (0.36)	0.39 (0.36)	0.39 (0.35)
Tone	0.40 (0.37)	0.41 (0.38)	0.41 (0.38)	0.40 (0.37)
$$\hbox {R}_1$$				
Binary	0.12 (0.14)	0.12 (0.14)	0.13 (0.14)	0.13 (0.15)
Polyphony	0.46 (0.37)	0.48 (0.38)	0.48 (0.38)	0.46 (0.37)
Intensity	0.48 (0.41)	0.47 (0.41)	0.51 (0.40)	0.52 (0.41)
Pause	0.40 (0.27)	0.39 (0.29)	0.41 (0.28)	0.41 (0.27)
Rhythm	0.37 (0.35)	0.37 (0.36)	0.39 (0.36)	0.39 (0.36)
Tone	0.38 (0.38)	0.38 (0.38)	0.40 (0.38)	0.39 (0.38)
$$\hbox {R}_1$$				
Binary	0.13 (0.14)	0.13 (0.16)	0.12 (0.14)	0.12 (0.14)
Polyphony	0.48 (0.38)	0.48 (0.36)	0.47 (0.36)	0.48 (0.36)
Intensity	0.49 (0.40)	0.52 (0.41)	0.50 (0.40)	0.49 (0.41)
Pause	0.40 (0.29)	0.44 (0.29)	0.44 (0.28)	0.43 (0.28)
Rhythm	0.38 (0.36)	0.40 (0.36)	0.39 (0.35)	0.39 (0.36)
Tone	0.39 (0.38)	0.40 (0.37)	0.39 (0.38)	0.39 (0.38)
$$\hbox {R}_1$$				
Binary	0.13 (0.14)	0.13 (0.16)	0.13 (0.14)	0.13 (0.13)
Polyphony	0.47 (0.37)	0.51 (0.37)	0.46 (0.36)	0.45 (0.36)
Intensity	0.50 (0.40)	0.52 (0.41)	0.49 (0.40)	0.49 (0.41)
Pause	0.41 (0.29)	0.46 (0.30)	0.41 (0.27)	0.42 (0.29)
Rhythm	0.39 (0.35)	0.42 (0.37)	0.38 (0.35)	0.38 (0.35)
Tone	0.38 (0.38)	0.38 (0.39)	0.41 (0.38)	0.39 (0.38)

Therefore, it may be deduced that the PSO agents’ movement is heavily affected by the inability of the underlying composition modules to compose music accurately. If the music composition modules composed music accurately, the features of the composed music would be “similar” to the ones that the respective agent requested. Although the term “similar” seems abstract, a quantification of the required “similarity” can be estimated by considering the error of the composed melodies, in comparison to the agents’ requests in Table 9. This error expresses the noisiness derived by the inaccuracy of the underlying music composition modules, which affects the fitness estimation and therefore the movement of each agent during the simulations. Nonetheless, even if the composition process is inaccurate, the system presents a converging behaviour which depends on the errors produced by the music composition modules.

**Fig. 10:**
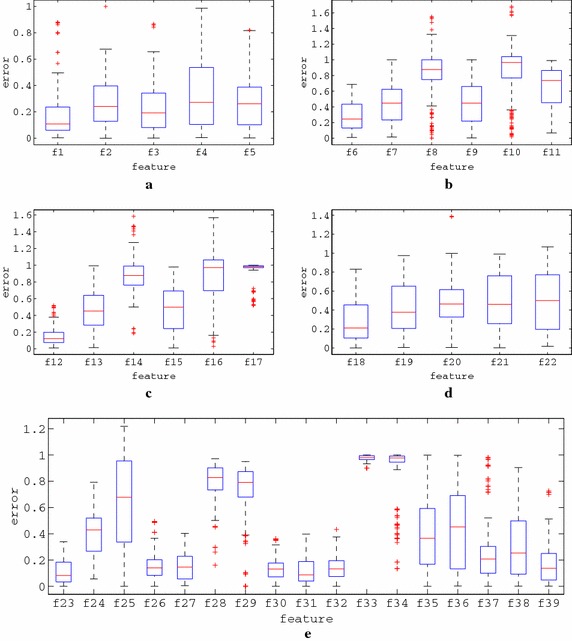
Box plots of the adaptation per feature between the composed music and the target features for the raters and setups that minimum error was achieved regarding rhythm (**a**–**d**) and tone (**e**). **a** $\hbox {R}_2\hbox {S}_1$ binary features. **b** $\hbox {R}_2\hbox {S}_1$ polyphony features. **c** $\hbox {R}_2\hbox {S}_1$ intensity features. **d** $\hbox {R}_2\hbox {S}_1$ pause features. **e** $\hbox {R}_2\hbox {S}_1$ tone features

**Fig. 11:**
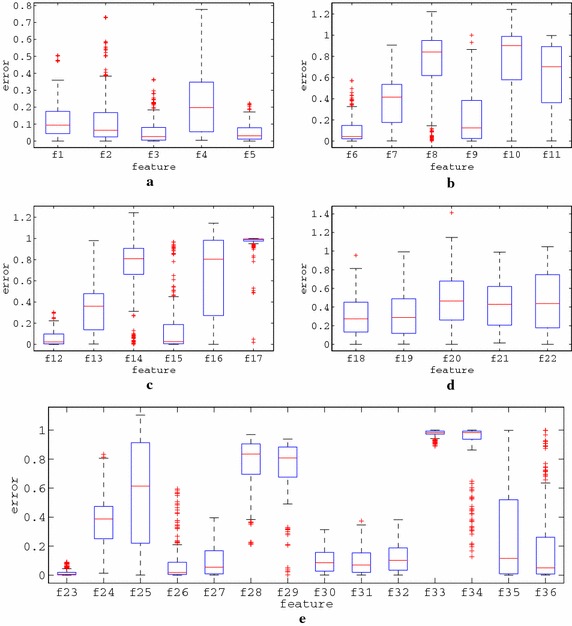
Box plots of the adaptation per feature between the composed music and the features requested by the agents in the last swarm evolution, for the raters and setups that minimum error was achieved regarding rhythm (**a**–**d**) and tone (**e**). **a** $\hbox {R}_2\hbox {S}_1$ binary features. **b** $\hbox {R}_2\hbox {S}_1$ polyphony features, **c** $\hbox {R}_2\hbox {S}_1$ intensity features. **d** $\hbox {R}_2\hbox {S}_1$ pause features, **e** $\hbox {R}_2\hbox {S}_1$ tone features

A deeper analysis on the error sources of the underlying music composition modules can be performed through the examination of the specific features that define their respective fitnesses. A shadowgraph of the system’s adaptivity at the level of music features is provided in Fig. 10, which illustrates the errors between the ideal features and the features of the four composed melodies in the last rating rounds of a representative rater–setup pair. The graphs of the remaining rater–setup pairs are similar. Therein, it is evident that some features exhibit systematically high error values, on contrast to some others. Moreover, Fig. 11 reveals that there is reciprocality in the errors between specific composition-to-agent and composition-to-ideal features. This fact further reveals the weaknesses of the underlying composition modules to adapt homogeneously to the melodic requirements of the agents, leading the system to overall suboptimal solutions. Features indexed from 37 to 39 in Fig. 10 are absent in the Fig. 11, since these features are passed from the agents to the tone module exactly as they are, in order to produce the list of available tones. Therefore, there is no matter of agent-to-melody adaptation regarding these three features.

The comparison of the overall errors that the system produced, according to the automatic rates’ ideal features, and the within-system errors, between the agent features and the composition features, led to a straight comparison of the distances between specific features themselves. Thereby, it is obvious that the underlying GA composition modules exhibit a “biased” optimisation behaviour, by minimising the error according to specific features, while ignoring the error produced by other features. Therefore, although the system is swiftly driven to locations that are closer to the ideal locations as examined in “Fitness convergence analysis” section, there are further potentialities for performance improvements by utilising more either more sophisticated music composition techniques, or by introducing more sophisticated (probably multi-objective) optimisation criteria.

## Conclusions

This work introduced a system that composes music automatically with evolutionary algorithms, in accordance to the rating provided by the user, who is provided with four melodies and rates them using her/his subjective criteria, giving rates on the rhythmic and tonal characteristics of these four melodies. These quadruples of melodies evolve to ones that encompass more pleasant content to the user both in terms of rhythm and tone. The system’s architecture incorporates a novel tow-level evolutionary scheme, with the higher level being based on the particle swarm optimisation (PSO) algorithm and the lower level on music composition modules that utilize genetic algorithms (GA). The agents’ positions on the higher level PSO describe musical characteristics in the form of music features, incorporating two sets of agents that describe two different music attributes, namely rhythm and tone. The fitness of each agent in the rhythm and tone swarms is provided by the listener, leading the agents in locations that encompass more promising musical characteristics for the user. The lower level GA modules compose music in accordance to the characteristics provided by each PSO agent, therefore composing music that is more pleasant to the listener as the PSO iterations progress.

The system is based on the PSO algorithm that constitutes a robust cornerstone. However, the underlying GA algorithms that are used for music composition are not guaranteed to compose music that is absolutely reflecting the characteristics that the PSO agents carry. Therefore, the produced melodies are expected to be placed on different locations in the feature space than the agents that are actually rated, introducing a noisy factor to the top level PSO system. Additionally, the ratings provided by a human user on the artistic content are expected to incorporate considerable uncertainty, introducing additional noise to the system. Therefore the robustness of the system under the imposition of noise by these factors was evaluated, with the utilisation of “artificial raters” that incorporated predefined music preferences, which also remained fixed throughout the rating rounds (PSO iterations). Although the choice to employ artificial raters for the experimental results sounds radical, in fact it was the only way to examine the convergence of the system; human users lack the ability to maintain a steady set of features throughout the simulations since they are affected by the musical content they are exposed to. To this end, four artificial raters were modeled in accordance to different human rating profiles, including different rating strictness scales and noise, which provide a “deterministic” means to examine the system’s robustness.

Exhaustive experimentation with multiple simulations over the system’s performance with all available artificial raters and four PSO setups yielded that the system is swiftly converging to the ideal features that the automatic rater desired. Two PSO setups were based on the standard PSO methodology, while the remaining two were based on a variation of PSO, the *rating-based* PSO (*r*-PSO), which was developed in the context of the presented work. In addition to system convergence, the diversity in musical characteristics throughout the rating rounds were examined, a fact that is important towards reducing the *user fatigue* phenomenon, which is common in interactive systems. Thereby, the *r*-PSO variation exhibited the ability to maintain a greater variability to the melodic content than the standard PSO throughout all iteration rounds and within each iteration round, while being equally or even more efficient than PSO towards fitness improvement. Finally, the system’s weaknesses were scrutinised, revealing that its overall performance depends on the music composition capabilities of the bottom level algorithms.

The primary contribution of the paper at hand is the presentation of system that performs feature evolution instead of melody evolution. In feature evolution, the user provides fitness through rating on the combination of features that are expressed by a melody. In fact, the user rates the features that are responsible for the generation of a melody, through the underlying music composition modules. As discussed in “Literature overview and motivation” section, the critical advantage of feature evolution is the fact that neighbouring points in the feature space describe melodies with similar musical characteristics, since several studies exhibited the potential of these features to categorize music according to aesthetic content, genre or composer among others. This locality coherence allows meaningful transitions of the agents on the top level, e.g. a point near a well-rated melody will most likely be a well-rated melody. Contrarily, the genotypical “neighbourhoods” are not coherent, in a sense that melodies with similar genotypes may be expressed by phenotypes that are distant in aesthetics, e.g. pleasantness and unpleasantness. Therefore, interactive music composition based on feature evolution could introduce a novel research direction where more robust interactive methodologies could be developed. Additionally, it should be noted that in the feature evolution setup that is tested, the notion *convergence* is not the same as the notion of *good music*, since the generated music is as good as efficient the features are.

The exhaustive study of the proposed system not only provides insights about the system in its current form, i.e. with the music composition submodules that have been described, but also introduces a methodological context to validate and compare potential future implementations. These implementations would be directed towards automatic music composition through feature evolution, not necessarily incorporating the presented setup [e.g. they could incorporate the Differential Evolution (Storn and Price 1997; Price et al. 2005) algorithm on the top level]. To this end, the utilisation of the artificial raters provides a solid framework for validating the convergence potential and the melodic capabilities of the system setup under examination. Thereby, the paper at hand has also contributed by proposing a methodology for assessing the robustness and the musical characteristics of an interactive feature-evolutionary music composition system. The latter fact is pivotal towards formulating interactive music composition systems in general, since the vast majority of evaluation methodologies rely solely on subjective tests, disallowing the potential of comparative studies (Pearce and Wiggins 2007).

## Footnotes

1 As a matter of fact, the European Research Committee has funded four large-scale research project around computational creativity from 2013 to 2016, namely Learning to Create (http://lrn2cre8.eu), Concept Creation Technology (http://conceptcreationtechnology.eu), Concept Invention Theory (http://www.coinvent-project.eu/en/home.html) and the What-If Machine (http://www.whim-project.eu).
